# Reciprocal compensation of caspase-2 and p53 regulates the DNA damage response in HCT116 colon cancer cells

**DOI:** 10.1016/j.jbc.2026.113199

**Published:** 2026-05-27

**Authors:** Karla E. Lopez, Leslie Bandera, Luiza E. Coutinho, Karen Zúñiga, Sarah Wright, Lisa Bouchier-Hayes

**Affiliations:** 1 Department of Pediatrics, Division of Hematology-Oncology, Baylor College of Medicine, Houston, Texas, USA; 2 William T. Shearer Center for Human Immunobiology, Texas Children’s Hospital, Houston, Texas, USA; 3 Department of Molecular and Cellular Biology, Baylor College of Medicine, Houston, Texas, USA

**Keywords:** caspase-2, cell cycle, DNA damage response, p53, homologous recombination

## Abstract

Caspase-2 has positive and negative effects on tumor growth in different cancer models. The mechanisms determining these dual outcomes of caspase-2 activation are unclear underscoring the need to assess caspase-2 function in different cell types. We report that high caspase-2 expression is associated with lower survival in patients with colon cancer and increased tumor size in HCT116 colon cancer xenografts. We investigated the interplay between caspase-2 and p53 in regulating the proliferation of these cells. While neither of these proteins had much of an effect on cell cycle arrest on their own, deficiency in both caspase-2 and p53 led to slower proliferation, prolonged G1 cell cycle arrest, and impaired homologous recombination. Caspase-2 loss resulted in increased p53 transcription and activation, while p53 loss resulted in increased caspase-2 activation suggesting that these two proteins compensate for each other. Strikingly, loss of caspase-2 led to disruption of p53-dependent transcription and enhanced the ability of p53 to bind to the promoter regions of the genes encoding mouse and human double minute 2 and p21. These results were unique to the HCT116 cells, but activation of the WNT pathway in RKO, a second colon cancer cell line, caused them to phenocopy HCT116 cells with respect to the requirement for caspase-2 and p53 for the DNA damage response. Our data support a model in which caspase-2 and p53 can compensate for the loss of each other to ensure cell cycle and homologous recombination proceed correctly. Together, our data suggest that inhibiting both proteins would be detrimental to colon cancer cell growth.

Caspase-2 is a member of the caspase family of proteases, many of which have essential roles in apoptosis (1). While caspase-2 is considered a pro-apoptotic protein, evidence suggests that it has important functions in regulating the cell cycle and the DNA damage response (DDR) (2). Loss of caspase-2 has been associated with aberrant tumor growth in a number of different murine cancer models (2, 3). Caspase-2 has shown tumor suppressive effects in murine models of some hematological cancers (Eμ-*Myc*-driven lymphoma (4) and *Atm* knockout-associated lymphoma (5)) and solid tumors (*Kras*-driven lung tumors (6) and MMTV-*c-neu*-driven mammary tumors (7)). However, caspase-2 has shown pro-oncogenic effects in TH-MYCN-induced neuroblastoma (8) and DEN-induced liver tumors (9). In each of these models, the absence of caspase-2 had minimal effects on apoptosis and there were reported differences in cell division and genomic instability. Therefore, it is likely that the role that caspase-2 plays in tumor growth is primarily through regulation of cell cycle events and genomic stability.

Similar to the protumor and antitumor effects of caspase-2 in different cancer types, the impact of caspase-2 on proliferation differs depending on the cell type. Loss of caspase-2 is associated with increased proliferation in transformed mouse embryonic fibroblasts (MEFs), and we previously reported that absence of caspase-2 was associated with a delayed exit of S phase, increased stalled replication forks, replication stress, and DNA damage in transformed MEFs and HeLa cells (4, 10). In contrast, in AML cells carrying a nucleophosmin 1 (NPM1) mutation, caspase-2 was proproliferative, and there were minimal differences in proliferation with or without caspase-2 in TH-MYCN neuroblastoma (8, 11). One element that could account for these differences is the status of *TP53*. While NPM1-mutated AML cells (OCI-AML3) and neuroblastoma express wildtype *TP53* (8, 12), HeLa and E1A/Ras transformed MEF cells have defective p53 pathways due to the expression of E6, which promotes the constant degradation of p53, and E1A that inhibits the transcriptional activity of p53, respectively (13, 14).

p53 is a tumor suppressor and transcription factor that is mutated in 50% of human cancers (15). It has multiple biological roles, and it is well-known for its role as a cell cycle checkpoint protein (16). Upon activation, p53 can induce apoptosis or cell cycle arrest and is a major regulator of the DDR (17). Its role in regulating the DDR is primarily through inducing G1 arrest allowing cells to repair DNA damage (18). In addition, p53 is involved in the pathways overseeing the repair of double-strand breaks (DSBs): nonhomologous end joining (NHEJ) and homologous recombination (HR). p53 has an indirect role in NHEJ by inducing cell death in the presence of unrepairable DNA damage (19, 20). In contrast, p53 can both positively and negatively regulate HR through transcriptional and nontranscriptional functions (21, 22, 23, 24, 25).

The requirement of p53 for caspase-2 function is not well understood, and there is conflicting evidence regarding the interplay between these two proteins. The upstream caspase-2 activator p53-induced death domain protein 1 (PIDD1) is a p53-inducible gene, but PIDD1 can be expressed in the absence of p53, and caspase-2 can be activated in the absence of PIDD1 (26, 27, 28). In addition, caspase-2 activation has been shown to be p53-independent in response to irradiation plus CHK1 inhibition in a zebrafish model (29) and following chromosomal missegregation in HCT116 colon cancer cells (30). The p53 negative regulator mouse and human double minute 2 (MDM2) is a caspase-2 substrate that, when cleaved, induces p53 activation and cell cycle arrest in response to chromosomal missegregation (31, 32). In addition, the absence of caspase-2 has been associated with downregulation of p53 target genes in MEF and MCF7 cells treated with DNA-damaging agents and in *Kras*-driven lung tumors (6, 7), placing p53 downstream of caspase-2 activation in these contexts. In contrast, p53 and p21 protein levels were unchanged in *Atm*^*−/−*^*/Casp2*^*−/−*^ lymphomas and TH-MYCN/*Casp2*^−/−^ neuroblastoma (5, 8). Therefore, the requirement of p53 for caspase-2 activation and its placement in the caspase-2 cascade differs depending on cell and tissue type. Due to the overlapping functions of caspase-2 with p53, it is important to understand the exact role of caspase-2 in modulating p53 function in different cell types and the impact of *TP53* status on caspase-2 activity. Here, we show that in the colon cancer cell line HCT116, which expresses wildtype *TP53*, caspase-2 restricts p53 activity to bypass arrest and facilitate HR and tumor growth.

## Results

### Low caspase-2 expression is associated with improved survival of patients with colorectal adenocarcinoma

**Figure 1:**
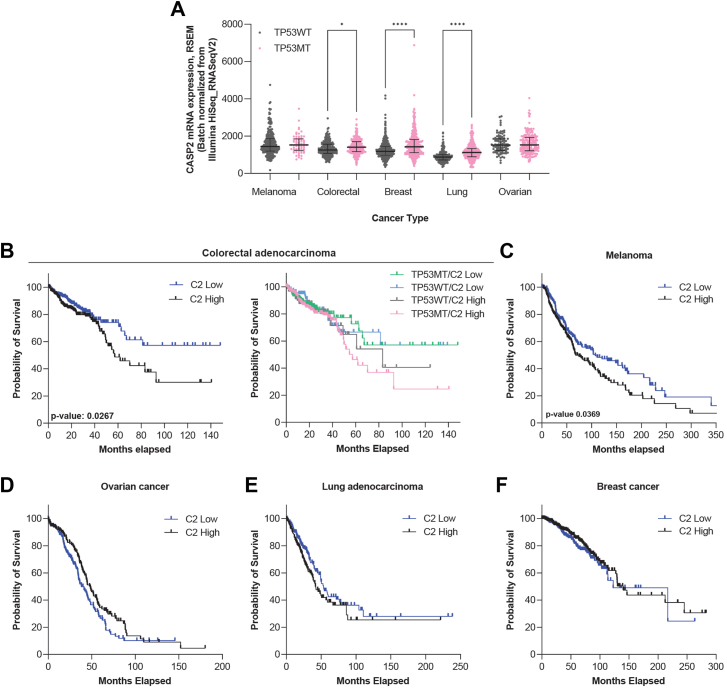
**Low expression of caspase-2 is associated with improved survival of patients with colorectal adenocarcinoma.***A, CASP2* mRNA expression of patients with skin cutaneous melanoma (TCGA, PanCancer Atlas) with wildtype *TP53* (n = 299) or mutated *TP53* (n = 64); colorectal adenocarcinoma (TCGA, PanCancer Atlas) with wildtype *TP53* (n = 211) or mutated *TP53* (n = 313); breast invasive carcinoma (TCGA, PanCancer Atlas) with wildtype *TP53* (n = 650) or mutated *TP53* (n = 344); lung adenocarcinoma (TCGA, PanCancer Atlas) with wildtype *TP53* (n = 432) or mutated *TP53* (n = 470); and ovarian serous cystadenocarcinoma (TCGA, PanCancer Atlas) with wildtype *TP53* (n = 98) or mutated *TP53* (n = 193); ∗*p* < 0.05, ∗∗∗∗*p* < 0.0001 (one-way ANOVA with Šídák's multiple comparison test). *B,* survival curve of patients with colorectal adenocarcinoma stratified by high (n = 247) and low *CASP2* mRNA levels (n = 253) (*left*) and high *CASP2* mRNA/*TP53* WT (n = 98), low *CASP2*/*TP53* WT (n = 100), high *CASP2/TP53*MT (n = 149), and low *CASP2/TP53*MT (n = 153). *C–F,* survival curves stratified by *CASP2* mRNA levels of patients with high *CASP2* (n = 171) or low *CASP2* (n = 177) with melanoma (*C*), patients with high *CASP2* (n = 145) or low *CASP2* (n = 145) with ovarian cancer (*D*), patients with high *CASP2* (n = 246) or low *CASP2* (n = 244) with lung adenocarcinoma (*E*), and patients with high *CASP2* (n = 489) or low *CASP2* (n = 492) with breast cancer (*F*). Significance in survival curve were calculated with the log-rank (Mantel–Cox) test.

To determine the association of *CASP2* expression and *TP53* mutation status in solid tumor, we interrogated the TCGA Pan Cancer Atlas in a number of tumor types (33, 34, 35). Because *CASP2* mutations are rare, we used *CASP2* expression as a readout. There was a significant increase in *CASP2* mRNA levels in the *TP53*-mutated tumors compared to the *TP53* wildtype tumors in breast cancer, lung adenocarcinoma, and colorectal adenocarcinoma, while there was no difference in melanoma or ovarian tumors (Fig. 1*A*). These data suggest that caspase-2 is enriched in the presence of inactivating *TP53* mutations in a subset of tumors.

Next, we assessed if the expression of *CASP2* affected the overall survival of patients. We stratified the patients into those with high *CASP2* expression and those with low *CASP2* expression. In patients with colorectal adenocarcinoma, low *CASP2* expression was associated with improved probability of survival (Fig. 1*B*). We further stratified this dataset into patients with wildtype or mutated *TP53* (Fig. 1*B*). Patients with lower expression of *CASP2* trended toward similar survival outcomes regardless of *TP53* status. In patients with high *CASP2* expression, *TP53* mutation caused some divergence from those with wildtype toward decreased probability of survival, but there were not sufficient patient numbers available in each group to reach significance. In the other cancer types, only melanoma showed a small but significant association between *CASP2* expression and decreased probability of patient survival (Fig. 1*C*), while there was no significant impact of *CASP2* expression on survival of patients with ovarian cancer (Fig. 1*D*), lung adenocarcinoma (Fig. 1*E*), or breast cancer (Fig. 1*F*).

### Caspase-2 levels determine tumor growth

**Figure 2:**
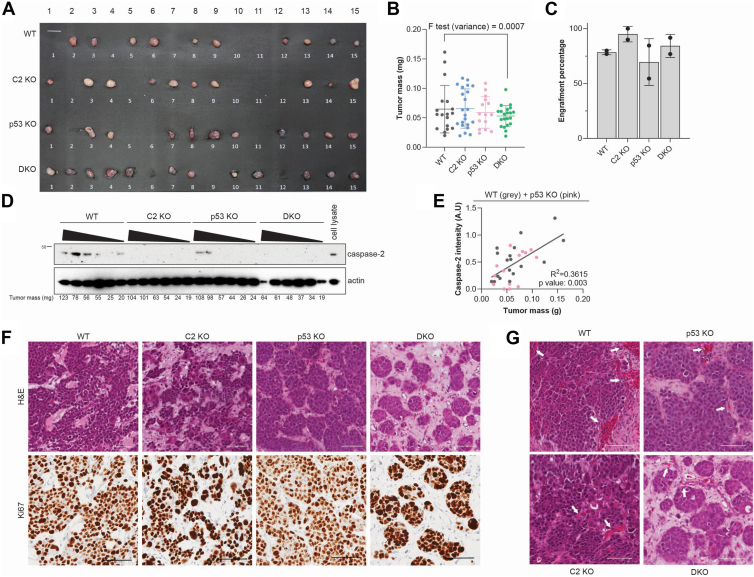
**Caspase-2 protein levels are associated with growth of engrafted HCT116 tumors.***A,* HCT116*TP53*^+/+^/scramble (WT), HCT116*TP53*^+/+^/CRISPR caspase-2 (C2 KO), HCT116*TP53*^−/−^/scramble (p53 KO), and HCT116*TP53*^−/−^/CRISPR caspase-2 (DKO) cells were injected into chorioallantoic membranes (CAMs), and tumors were harvested 1 week later. All the tumors from the first experiment are shown and are representative of two independent experiments. *Spaces* indicate failure to engraft. Bar, 1 cm. *B,* tumor mass was measured 1 week postengraftment. Each point on the graph represents a single tumor collected over two independent experiments with error bars representing standard deviation. Variance was calculated with by F test with *p* = 0.0007. *C,* the percentage of engraftment for each genotype is shown. Results are the average of two independent experiments plus or minus standard deviation. *D,* representative lysates of WT, C2 KO, p53 KO, and DKO tumors were immunoblotted for caspase-2 with actin as a loading control. Samples were run in order of tumor mass, largest to smallest, with the mass of each tumor indicated below. *E,* the mass of each WT and p53 KO tumor was plotted against relative intensity of caspase-2 as measured by densitometry of western blots. *F,* representative images of hematoxylin and eosin (H&E) (*upper*) and Ki-67 staining (*lower*) of tumor sections. Bar, 60 μm. *G,* representative images of vasculature of CAM tumors. *Arrows* indicate vascular regions. Bar, 60 μm.

Given that we saw the most significant association between survival and caspase-2 expression levels in colon cancer cells, we next investigated if caspase-2 has a p53-dependent effect on tumor growth in colon cancer cells. We used the colon cancer cell line HCT116 because it has an intact DDR and spindle-dependent checkpoints, and because a derivative of these cells, where both *TP53* alleles were disrupted by targeted homologous recombination, have been well characterized (36, 37, 38, 39). We used CRISPR/Cas9 to delete caspase-2 from HCT116*TP53*^+/+^ and HCT116*TP53*^−/−^ cells. We used the chorioallantoic membrane (CAM) xenograft assay because it allowed us to engraft and grow tumors in a fast and cost-efficient manner compared to mice (40). Thus, we investigated the ability of HCT116*TP53*^+/+^/CRISPR control (WT), HCT116*TP53*^+/+^/CRISPR caspase-2 (C2 KO), HCT116*TP53*^−/−^/CRISPR control (p53 KO), and HCT116*TP53*^−/−^/CRISPR caspase-2 (DKO) cells to engraft and grow on the CAM. One week following engraftment, we harvested the tumors and measured their mass (Fig. 2, *A* and *B*). The WT, C2 KO, and p53 KO cells grew into tumors of varying tumor sizes with similar ranges for each. The p53/C2 DKO cells trended toward producing smaller tumors, but this was not significant compared to the other genotypes. Of note, the range of tumor size was significantly smaller for the p53/C2 DKO cells compared to the other genotypes (Fig. 2*B*). The rate of engraftment was similar across the cell lines. (Fig. 2*C*). To determine if there was any correlation between tumor mass and caspase-2 expression, we measured the protein levels of caspase-2 by Western blot (Fig. 2*D*). Consistent with the human data, we noted a positive correlation between caspase-2 expression and tumor mass in WT and p53 KO tumors, where larger tumors tended to have higher levels of caspase-2 (Fig. 2*E*). We could not measure any detectable p53 protein in the tumors, so we were unable to do a similar correlative analysis for p53. These data suggest that lower levels of caspase-2 restrict growth of tumors derived from HCT116 cells, likely accounting for the homogeneity of tumor mass in the p53/C2 DKO tumors.

Histological analysis showed that the p53/C2 DKO tumor cells were arranged in a clustered fashion surrounded by stroma. In contrast, the wildtype, C2 KO, and p53 KO tumors grew in a more diffuse pattern with less stroma visible (Fig. 2*F*). This clustering of the tumor cells was confirmed by Ki-67 staining showing the dividing cells only in the tumor regions. Compared to the other genotypes, there were fewer Ki-67-positive cells in the absence of both caspase-2 and p53, suggesting a lower proliferation rate. There was also evidence of increased vascularity in the wildtype group compared to the knockout groups (Fig. 2*G*). The tumor cell clustering and decreased blood supply to the p53/C2 DKO tumors may explain their more homogenous tumor size compared to the other genotypes. Together, these data suggest that in HCT116 colon cells, caspase-2 and p53 function to promote tumor growth by enhancing proliferation.

### Caspase-2 compensates for p53 to regulate the G1 checkpoint

**Figure 3:**
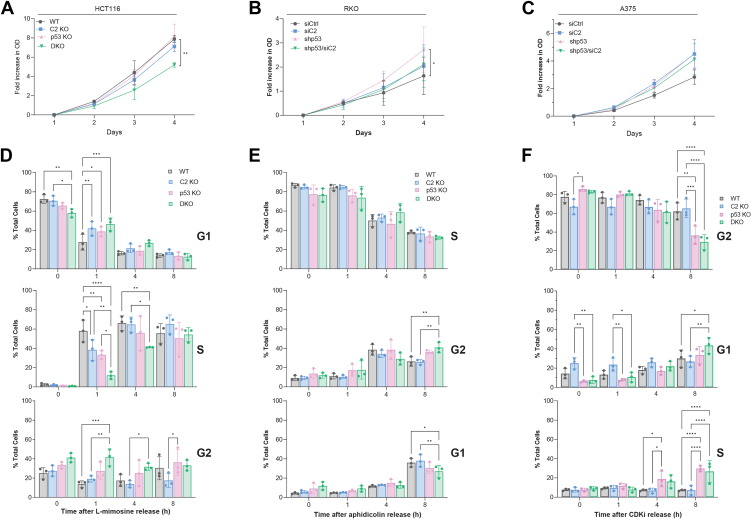
**Caspase-2 and p53-deficient cells have a delayed exit from G1 arrest.***A–C,* cells of each genotype were seeded at 1.25 × 10^3^ cells/well and cell viability was measured by MTT at the indicated times in normal cycling HCT116*TP53*^+/+^/CRISPR scramble (WT), HCT116*TP53*^+/+^/CRISPR caspase-2 (C2 KO), HCT116*TP53*^−/−^/CRISPR scramble (p53 KO), and HCT116*TP53*^−/−^/CRISPR caspase-2 (DKO) cells (*A*), RKO cells (*B*) or A375 cells (*C*) stably expressing an shRNA targeting p53, transfected with either siRNA against caspase-2 or a control siRNA (25 nM). Results are the average of three independent experiments plus or minus standard deviation. ∗*p* < 0.05, ∗∗*p* < 0.01 (two-way ANOVA with Dunnett’s multiple comparison test, compared to WT or siCtrl). *D–F,* HCT116 WT, C2 KO, p53 KO, and DKO cells were treated with L-mimosine (0.5 mM) for 22 h (*D*), aphidicolin (1 μM) for 16 h (*E*), and double thymidine block followed by inhibition of CDK1 using Ro-3306 (CDK1i, 10 μM) for 12 h (*F*). Following arrest, the treatment was removed and replaced with fresh media (0 h). Cells were pulsed with BrdU (10 μM) for 30 min prior to each time point and then stained with anti-BrdU-FITC/7-AAD at the indicated time points. The proportion of cells in S-, G1-, and G2-phase was determined by flow cytometry. The top panel in each case shows the cell cycle stage in which arrest occurred. Results are the average of three independent experiments plus or minus standard deviation. ∗*p* < 0.05, ∗∗*p* < 0.01, ∗∗∗*p* < 0.001, and ∗∗∗∗*p* < 0.0001 (two-way ANOVA with Tukey’s multiple comparison test).

Given the positive association of caspase-2 expression with tumor size, we next determined the rate of proliferation in HCT116 cells using an MTT viability assay. The absence of caspase-2 and p53 on their own had no effect on the increase in number of viable cells over a 4-day period compared to the wildtype cells (Fig. 3*A*). Surprisingly, loss of both caspase-2 and p53 resulted in a significant decrease in viability. To confirm that this decrease in viability was due to the differences in proliferation and not the result of increased cell death, we measured apoptosis. As expected, loss of p53 completely protected against apoptosis under these conditions in the presence and absence of caspase-2 (Fig. S1*A*). Loss of caspase-2 alone had minimal impact on apoptosis. Therefore, differences in apoptosis alone cannot explain the lower growth rate in absence of both caspase-2 and p53, suggesting that these proteins cooperate to promote cell proliferation. To explore the generality of this phenomenon, we also measured proliferation of RKO cells, a second colon cancer cell line, and the melanoma cell line A375, both of which also expresses wildtype *TP53* (41, 42). We were unable to derive CRISPR-mediated caspase-2 deletions from either of these cell lines, so we stably expressed a shp53 to knockdown p53 and used siRNA to silence caspase-2 (Fig. S1, *B* and *C*). In contrast to the HCT116 cells, and consistent with previous reports in other cell types (4, 10), loss of caspase-2 alone led to a slight increase in cell proliferation in RKO and A375 cells (Fig. 3, *B* and *C*) that was not impacted by the concomitant loss of p53. In fact, in RKO cells, only the p53-deficient cells had a significantly higher rate of proliferation.

Because HCT116 cells show a unique pattern of reduced growth in the absence of caspase-2 and p53, we decided to further characterize the cell cycle in these cells. We synchronized cells at each stage of the cell cycle and tracked the effect of loss of p53 and/or caspase-2 on recovery from arrest. First, we induced G1-arrest with the compound L-mimosine. Following arrest, we allowed the cells to recover after washout of the drug. While the wildtype, C2 KO, and p53 KO cells arrested in G1 to a similar extent, the p53/C2 DKO cells had a significant reduction in the number of cells arrested at G1 compared to the wildtype and C2 KO cells (Figs. 3*D* and S1*D*). This result suggests that the G1 checkpoint that is bypassed in the absence of p53 is rescued by caspase-2 in these cells. Following the release of HCT116 cells from G1 arrest, the wildtype cells re-entered the cycle again, as detected by a steady decrease in the proportion of cells in G1 over time. This was accompanied by a rapid increase in the proportion of cells in S-phase followed by a slower increase in G2-phase cells (Fig. 3*D*). In the absence of caspase-2 or p53, there was a significant delay in the initial transition from G1 to S, but by 4 h, the cells had caught up with the wildtype population. In contrast, the p53/C2 DKO showed a dramatic impairment in recovery from G1 arrest, with the majority of cells persisting in G1 and minimal cells moving into S-phase. These cells took a full 8 h to catch up with the other three genotypes. This result suggests that, DKO cells have delayed in recovery from G1 arrest.

Next, we arrested the cells in S-phase. We arrested WT, C2 KO, p53 KO, and p53/C2 DKO HCT116 cells with aphidicolin and then allowed recovery by washing out the drug (Figs.3E and S1*E*). We observed similar levels of S-phase arrest across all genotypes. Unlike the response to G1 arrest, the recovery of these cells was much slower and was not seen until the 4 h time point. The main differences between the genotypes were detected 8 h following arrest when the transition from S to G2 phase was accelerated in the p53/C2 DKO cells compared to wildtype cells. Phase transition was similarly accelerated in p53 KO cells, but this was not significant, while C2 KO cells were similar to the wildtype cells. We previously reported a delayed exit in S-phase and increased proliferation in caspase-2-deficient MEF (10). The opposite effect on S-phase exit in the absence of caspase-2 and p53 that we observe in HCT116 cells is thus consistent with the lower proliferation rates.

Finally, we arrested HCT116 cells with a CDK1 inhibitor. In response to CDK1 inhibition, the recovery of C2 KO cells was similar to the WT cells and, if anything, appeared to have a prolonged G1 and G2 arrest. The p53 KO and p53/C2 DKO cells both recovered from G2 arrest at similar increased rates compared to the wildtype cells (Figs. 3*F* and S1*F*). Overall, it appears that recovery from G2 arrest is primarily p53 dependent. Taken together, these results suggest that p53 and caspase-2 can compensate for the loss of each other to control the G1 checkpoint in HCT116 cells.

### Caspase-2 and p53 cooperate to regulate DNA repair

**Figure 4:**
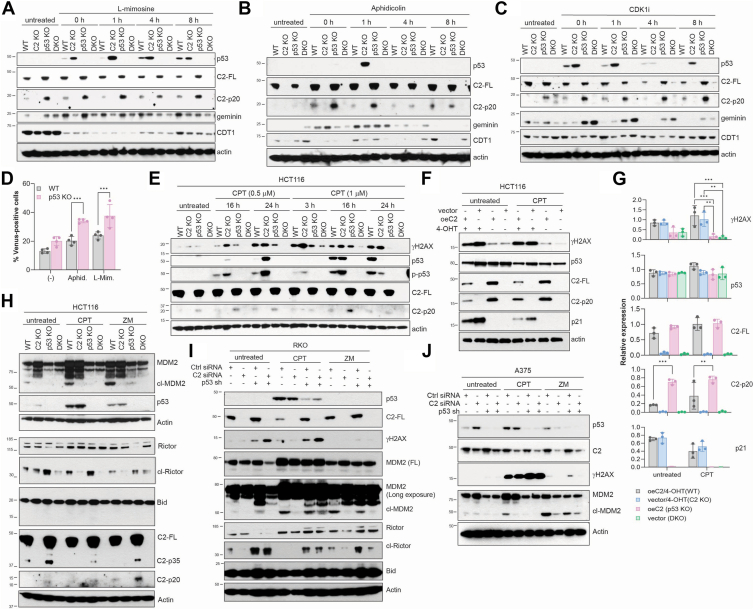
**Loss of caspase-2 increases p53 activation and loss of p53 increases caspase-2 activation in HCT116 cells.***A–C,* HCT116*TP53*^+/+^/CRISPR scramble (WT), HCT116*TP53*^+/+^/CRISPR caspase-2 (C2 KO), HCT116*TP53*^−/−^/CRISPR scramble (p53 KO), and HCT116*TP53*^−/−^/CRISPR caspase-2 (DKO) cells were left untreated or treated with L-mimosine (0.5 mM) for 22 h (*A*), aphidicolin (1 μM) for 16 h (*B*), and double thymidine block followed by inhibition of CDK1 using Ro-3306 (CDK1i, 10 μM) for 12 h (*C*) followed by replacement of fresh media (0 h). Cell lysates were collected at the indicated time points after recovery and were immunoblotted for the indicated proteins with actin as a loading control. *D,* HCT116 WT and HCT116*TP53* p53 KO cells were transfected with pBiFC.C2-Pro VC155 (10 ng), pBiFC.C2-Pro VN173 (10 ng), and dsRedmito (10 ng) as a transfection reporter. The cells were left untreated or treated with aphidicolin (Aphid, 1 μM) or L-mimosine (L-mim, 1 mM). The next day, the percentage of Venus-positive transfected cells was determined for at least 200 cells per treatment. Results are the average of three independent experiments plus or minus standard deviation. ∗∗∗*p* < 0.001 (Unpaired *t* test with Welch's correction). *E,* WT, C2 KO, p53 KO, and DKO cells were *left* untreated or treated with the indicated amounts of camptothecin (CPT). After 3 h, the treatment was removed and replaced with fresh media (0 h). Cell lysates were collected at the indicated time points after recovery and were immunoblotted for the indicated proteins with actin as a loading control. *F,* DKO cells stably expressing pMX-p53ERtam-IRES-GFP were transfected with pcDNA3.1 empty vector (vector) or pcDNA3.1 caspase-2 (oeC2). The following day, the cells were treated with tamoxifen (4-OHT, 50 nM) and CPT (0.5 μM) for 3 h followed by a 24 h recovery. The lysates were immunoblotted for the indicated proteins. *G,* band intensity of the proteins in (*F*) was quantified and normalized over the loading control actin. ∗∗*p* < 0.01, ∗∗∗*p* < 0.001 (two-way ANOVA with Tukey’s multiple comparison test). *H,* HCT116 WT, C2 KO, p53 KO, and DKO cells were left untreated or treated with CPT (0.5 μM) for 3 h followed by replacement with fresh media for 24 h or with ZM447439 (ZM, 2 μM) for 16 h. Cell lysates were immunoblotted for the indicated proteins with actin as a loading control. *I–J,* RKO (*I*) or A375 (*J*) cells stably expressing an shRNA targeting p53 were transfected with either siRNA against caspase-2 or a control siRNA (25 nM). Two days after transfection, cells were treated as in (*H*), and cell lysates were immunoblotted for the indicated proteins with actin as a loading control. Each western blot result shown is representative of three independent experiments. See Figs. S2 and S3 for quantification of protein levels in each panel.

Next, we measured caspase-2 and p53 activation following cell cycle arrest at G1, S, and G2 in HCT116 cells deficient in caspase-2, p53, or both. We measured p53 activation by p53 protein stabilization and used caspase-2 cleavage, as indicated by the appearance of its p20 subunit, as an indicator of caspase-2 activation (Figs. 4, *A*–*C* and S2, *A*–*C*). Interestingly, following arrest in G1 with L-mimosine, p53 stabilization was consistently and significantly increased in the absence of caspase-2 compared to WT cells at the 0 to 4 h timepoints (Figs. 4*A* and S2*A*). Similarly, in cells treated with aphidicolin to induce S arrest or a CDK1 inhibitor to induce G2 arrest, p53 levels were significantly increased but not at all time points (Figs. 4, *B*, *C* and S2, *B*, *C*). In p53 KO cells following G1 and G2 arrest, caspase-2 cleavage, as measured by appearance of the p20 subunit, was consistently and significantly increased in the absence of p53 but not increased to the same extent following S arrest. Because caspase-2 cleavage can occur in the absence of activation (43), we confirmed the increase of caspase-2 activation using the bimolecular fluorescence complementation (BiFC) caspase-2 reporter. This reporter measures oligomerization of caspase-2 prodomains fused to nonfluorescent fragments of Venus that refold and fluoresce when they undergo induced proximity, the proximal step in caspase-2 activation (44). We measured caspase-2 BiFC in the presence or absence of p53 after arrest in G1 and S using L-mimosine and aphidicholin, respectively, and caspase-2 activity was increased in the absence of p53 in both cases (Fig. 4*D*). Together, these data suggest that caspase-2 negatively regulates p53 activation and that p53 negatively regulates caspase-2 activation following cell cycle arrest.

To determine if this reciprocal relationship between p53 and caspase-2 activation has any impact on the DNA damage response, we used camptothecin (CPT), a topoisomerase I inhibitor that induces apoptosis, cell cycle arrest, and DNA damage. We treated HCT116 WT, C2 KO, p53 KO, and DKO cells with CPT for 3 h followed by washout and recovery for up to 24 h. Similar to the response to the cell cycle arrest inducers, in the absence of caspase-2, we observed earlier and increased p53 activation, as measured by p53 protein stabilization and by phosphorylation at Serine 15, a known p53 activation marker (45). We also observed an increase in caspase-2 cleavage in the absence of p53, compared to wildtype cells (Figs. 4*E* and S2*D*). We investigated the impact of loss of p53 on the accumulation of DSBs by probing for phosphorylation of H2AX (γH2AX), a DNA repair protein that translocates to DSBs (46). While there was a trend toward increased γH2AX levels in C2 KO cells compared to WT at some timepoints, these differences were not significant across multiple experiments. However, the absence of p53 led to accelerated clearance of γH2AX. Surprisingly, in the absence of both p53 and caspase-2, γH2AX was almost absent. We confirmed this result by immunostaining cells for γH2AX (Fig. S2*E*). To confirm that this phenotype was specifically due to the absence of caspase-2 and p53, we stably expressed p53ER^TAM^ in the HCT116 p53/C2 DKO cells. p53ER^TAM^ consists of a wildtype *TP53* gene fused to a modified binding ligand domain of the estrogen receptor that is only functional in the presence of tamoxifen (47, 48). The p53 target p21 was increased in cells treated with tamoxifen, confirming the induction of p53 (Fig. 4, *F* and *G*). We replaced caspase-2 by transiently expressing caspase-2 in the HCT116 DKO p53ER^TAM^ cells. Transfected caspase-2 is activated upon expression as noted by the appearance of the p20 active subunit. Induction of both caspase-2 and p53 (WT equivalent) induced γH2AX without treatment. Following treatment with CPT, cells expressing both caspase-2 and p53 had the highest expression of γH2AX mimicking the phenotype we observed in the WT CPT-treated cells in Figure 4*E*. Compared to the WT equivalent cells, cells with induced p53 but no caspase-2 expression (C2 KO equivalent) had similar γH2AX levels, and p53 activity was increased as measured by an increase in p21 expression. In the cells overexpressing caspase-2 without p53 induction (p53 KO equivalent), we observed an increase in both full-length caspase-2 and the cleavage fragment compared to the WT equivalent cells with and without treatment. As before, cells transfected with the vector (DKO equivalent) had no induction of γH2AX. These data indicate that differences in these cells are due to the loss of caspase-2 and/or p53 and not any other genetic difference.

To determine how caspase-2 could be regulating γH2AX and p53 activation, we probed for the cleavage of three known caspase-2 substrates: the p53 negative regulator, MDM2, the proapoptotic protein, BID, and the mTORC2 component, Rictor (11, 31, 49, 50). As a control, we treated the cells with the Aurora B kinase inhibitor ZM447439 (ZM), which induces cytokinesis failure and robust MDM2 cleavage in a caspase-2-dependent manner (32). In HCT116 cells, we observed caspase-2-dependent cleavage of MDM2 and Rictor following CPT and ZM treatment but no detectable cleavage of BID (Figs. 4*H* and S3*A*). In HCT116 C2 KO cells treated with CPT, p53 stabilization was increased despite the lower MDM2 cleavage suggesting that the increase in p53 activation in the absence of caspase-2 is unlikely to be a result of impaired MDM2 function. Interestingly, Rictor cleavage increased in the absence of p53 in a caspase-2-dependent manner, suggesting that blocking Rictor cleavage by caspase-2 may contribute to increased p53 activation.

In RKO and A375 cells, p53 activation and γH2AX levels following CPT treatment were similar in the presence and absence of caspase-2, and γH2AX was significantly increased in the absence of p53 with or without caspase-2 (Figs. 4, *I*, *J* and S3, *B*, *C*). MDM2 was cleaved in a caspase-2-dependent manner in both cell types. In RKO cells, there was no BID cleavage, and while we observed Rictor cleavage, it was predominantly cleaved in the absence of p53, with minimal caspase-2 dependence. Thus, even in cells with normal p53 function, the roles of caspase-2 can differ dramatically. The results in RKO and A375 cells are consistent with the different proliferation profiles compared to the HCT116 cells (Fig. 3, *A*–*C*).

### Caspase-2 and p53 cooperate to regulate homologous recombination

**Figure 5:**
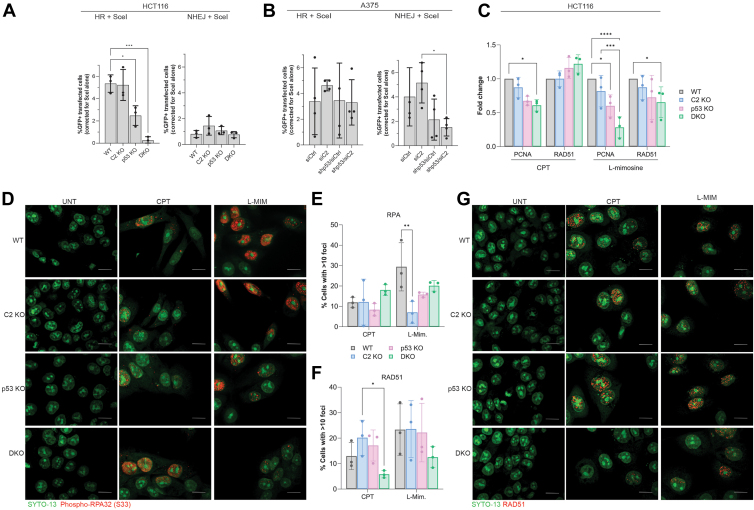
**Combined loss of caspase-2 and p53 impairs DNA repair by homologous recombination.***A,* HCT116*TP53*^+/+^/CRISPR scramble (WT), HCT116*TP53*^+/+^/CRISPR caspase-2 (C2 KO), HCT116*TP53*^−/−^/CRISPR scramble (p53 KO), and HCT116*TP53*^−/−^/CRISPR caspase-2 (DKO) cells stably expressing pDRGFP (HR, *left*) or pimEJ5GFP (NHEJ, *right*) were transiently transfected with or without I-SceI (100 ng) and with H2B-mCherry (150 ng) as a transfection reporter. Three days after the transfection, the percentage of mCherry cells expressing GFP was determined by flow cytometry. Results are the average of three independent experiments plus or minus standard deviation. ∗*p* < 0.05, ∗∗∗*p* < 0.001 (one-way ANOVA with Dunnett’s multiple comparison test). *B,* A375 cells stably expressing a shp53 and pDRGFP (HR, *left*) or pimEJ5GFP (NHEJ, *right*) were transiently transfected with I-SceI (100 ng), H2B-mCherry (150 ng) as a transfection reporter, and either siRNA against caspase-2 or a control siRNA (25 nM). Three days after transfection, the percentage of mCherry cells expressing GFP was determined by flow cytometry. Results are the average of three independent experiments plus or minus standard deviation. ∗*p* < 0.05 (one-way ANOVA with Dunnett’s multiple comparison test). *C,* HCT116 WT, C2 KO, p53 KO, and DKO cells were left untreated or treated with camptothecin (CPT, 0.5 μM) for 3 h followed by replacement with fresh media for 24 h or with L-mimosine (0.5 mM) for 22 h. Gene expression of *PCNA* and *RAD51* was measured by real-time PCR. Results are the average of three independent experiments plus or minus standard deviation. ∗*p* < 0.05, ∗∗∗*p* < 0.001, ∗∗∗∗*p* < 0.0001 (Two-way ANOVA with Tukey’s multiple comparison test). *D–G,* HCT116 WT, C2 KO, p53 KO, and DKO cells were treated as in (*C*) and stained for phospho-RPA32 (S33) (*D*, *E*) or RAD51 (*F*, *G*) and SYTO-13 to stain nuclei. Representative images show nuclei in *green* with phospho-RPA32 (S33) in *red* (*D*) or RAD51 in *red* (*G*). Bar, 10 μm. Image quantitation of the images is shown in (*E*, *F*). The percentage of cells with ≥ 10 RAD51 and phospho-RPA32 (S33) foci were counted from 25 different images per treatment (∼300 cells). Results are the average of three independent experiments plus or minus standard deviation. ∗*p* < 0.05, ∗∗*p* < 0.01, ∗∗∗*p* < 0.001, ∗∗∗∗*p* < 0.0001 (Two-way ANOVA with Tukey’s multiple comparison test). HR, homologous recombination; NHEJ, nonhomologous end joining.

The rapid phosphorylation of H2AX detects the presence of DSBs in cells (46), which makes the DNA more accessible so it can be recognized by downstream proteins that repair the DNA (51). Therefore, the reduction of γH2AX we see in the absence of both p53 and caspase-2 may be the result of less DNA damage or could indicate an impaired DNA repair response preventing its phosphorylation and translocation to DSBs. To directly measure DNA repair, we stably expressed two DNA repair reporters in each cell line that measure the efficiency of DNA repair by HR or NHEJ. Under normal conditions, these reporters do not express GFP (52, 53). In the HR reporter, expression of the endonuclease I-SceI cleaves one of two defective GFP repeats, and the cleaved DNA is repaired by HR. In the NHEJ reporter, two I-SceI sites in the reverse direction remove a region between GFP and its promoter producing DSBs with incompatible ends that are repaired by NHEJ. Although p53 is known to repress HR (25), in HCT116 cells expressing the HR reporter, repair was reduced in the absence of p53. Strikingly, HR was almost completely ablated in the absence of both caspase-2 and p53. Loss of caspase-2 alone had minimal effects on the levels of HR (Fig. 5*A*). In contrast, in HCT116 cells expressing the NHEJ reporter, we observed similar efficiency of repair across the genotypes consistent with an indirect role of p53 in NHEJ (Fig. 5*A*). This result suggests that p53 and caspase-2 cooperate to regulate DNA repair by HR in HCT116 cells. In contrast, in A375 cells, loss of caspase-2 and p53 had no significant impact on DNA repair by HR, and NHEJ was decreased to similar extents in the absence of p53 alone or the combined absence of p53 and caspase-2 (Figs. 5*B* and Fig. S4). Therefore, in A375 cells, NHEJ appears to be p53 dependent with caspase-2 having minimal effects on repair. These cells are reported to have lower expression of HR proteins and are likely more NHEJ dependent (54, 55, 56).

To confirm these results, we measured the expression of the two DNA repair genes required for HR, proliferating cell nuclear antigen (*PCNA)*, and *RAD51* (57, 58). In p53/C2 DKO cells compared to WT cells, there was a significant decrease in *PCNA* expression following CPT and L-mimosine and a significant decrease in *RAD51* expression following L-mimosine but not CPT treatment (Fig. 5*C*). *PCNA* expression was reduced to an intermediate level after L-mimosine treatment in the absence of p53, while loss of p53 alone had minimal effects on *RAD51* expression.

Next, we determined the impact of caspase-2 and p53 in DNA resection formation during HR by measuring recruitment of phospho-RPA32 (S33) to the DNA damage sites (59, 60). We observed a large decrease in phospho-RPA32 (S33) recruitment in the absence of caspase-2 after treatment with L-mimosine, but no difference in recruitment following CPT treatment across the genotypes (Fig. 5, *D* and *E*). This result suggests that caspase-2, and not p53, is important for recruitment of phospho-RPA32 (S33) during cell cycle arrest. Following RPA binding to ssDNA, RAD51 displaces RPA to promote homology search and strand exchange (57, 61, 62, 63, 64, 65). Thus, we measured RAD51 recruitment to the DNA damage sites (Fig. 5, *F* and *G*). After CPT treatment, loss of caspase-2 resulted in a slightly increased recruitment of RAD51 which was decreased to below wildtype levels in the absence of both caspase-2 and p53 (Fig. 5, *F* and *G*). There was a similar decrease in RAD51 recruitment in the p53/C2 DKO cells after L-mimosine treatment, but this did not reach statistical significance. Together, these data suggest that the decrease in γH2AX in the absence of caspase-2 and p53 in HCT116 cells is not due to increased DSBs but to impaired HR. In addition, the earlier step in HR, RPA recruitment is caspase-2 dependent, while the recruitment of RAD51 requires both caspase-2 and p53.

### Activation of β-catenin enhances the ability of caspase-2 and p53 to regulate the DNA damage response

**Figure 6:**
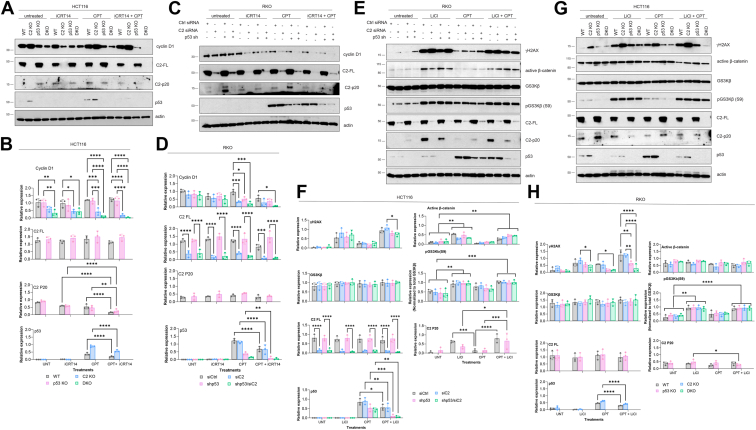
**Modulation of β-catenin signaling facilitates caspase-2 and p53 reciprocal compensation.***A*, HCT116*TP53*^+/+^/CRISPR scramble (WT), HCT116*TP53*^+/+^/CRISPR caspase-2 (C2 KO), HCT116*TP53*^−/−^/CRISPR scramble (p53 KO), and HCT116*TP53*^−/−^/CRISPR caspase-2 (DKO) cells were left untreated or treated with camptothecin (0.5 μM, CPT), iCRT14 (10 μM), or both for 3 h followed by replacement with fresh media or iCRT14 (10 μM) for 24 h. Cell lysates were immunoblotted for the indicated proteins with actin as a loading control. *B,* protein quantification of blots in (*A*). *C,* RKO cells stably expressing an shRNA targeting p53 were transfected with either siRNA against caspase-2 or a control siRNA (25 nM) and treated as in (*A*). Cell lysates were immunoblotted for the indicated proteins with actin as a loading control*. D,* protein quantification of blots in (*C*). *E,* The indicated RKO cells were left untreated or treated with CPT (0.5 μM), LiCl (20 mM), or both for 3 h followed by replacement with fresh media or LiCl (20 mM) for 24 h. Cell lysates were immunoblotted for the indicated proteins with actin as a loading control. *F,* protein quantification of blots in (*E*). *G,* HCT116 WT, C2 KO, p53 KO, and DKO cells were treated as in (*E*). Cell lysates were immunoblotted for the indicated proteins with actin as a loading control. *H,* protein quantification of blots in (*A*). Results are the average of three independent experiments plus or minus standard deviation. ∗*p* < 0.05, ∗∗*p* < 0.01, ∗∗∗*p* < 0.001, ∗∗∗∗*p* < 0.0001 (Two-way ANOVA with Tukey’s multiple comparison test). LiCl, lithium chloride.

Given that the RKO and HCT116 cell lines correspond to the same cancer type and are both wildtype for p53, we aimed to understand the reason for the phenotypic differences between cells. The co-activator of the WNT pathway, BCL9L, has been shown to regulate caspase-2 expression in colon cancer (30), and we reported that in NPM1c+ AML cells, loss of caspase-2 impairs WNT signaling (11). Interestingly, while RKO cells express wildtype *CTNNB1,* the gene that encodes the WNT pathway transcriptional co-activator β-catenin, HCT116 cells harbor a heterozygous mutation in the *CTNNB1* gene resulting in a constituently active WNT pathway (66, 67). Thus, we hypothesized that the β-catenin mutation contributes to the phenotypic differences between RKO and HCT116 cells lines with respect to caspase-2 function. To test this hypothesis, we treated HCT116 cells and RKO cells with the β-catenin inhibitor, iCRT14, which prevents the interaction between β-catenin and its target T-cell factor (68). Inhibition of the WNT pathway blocked the stabilization of p53 in C2 KO cells and reduced cleavage of caspase-2 in p53 KO cells (Fig. 6, *A* and *B*), but caspase-2 loss did not have any impact on p53 stabilization in RKO cells in which β-catenin is not active (Fig. 6, *C* and *D*). These results suggest that the reciprocal compensation between caspase-2 and p53 is mediated by β-catenin. Cyclin D1 is a direct target gene for β-catenin (69). In HCT116 cells, cyclin D1 expression was completely blocked in the absence of both caspase-2 and p53 in response to iCRT14- and CPT-treated cells, similar to caspase-2- and p53-deficient RKO cells treated with CPT alone. Thus, blocking β-catenin in HCT116 cells changes the CPT-induced expression pattern of cyclin D1 to be more similar to that seen in RKO cells.

We next investigated if activating β-catenin in RKO cells converted them to behave more like HCT116 cells. We treated both cell types with lithium chloride (LiCl) to induced GS3Kβ phosphorylation at serine 9, resulting in its inhibition, permitting β-catenin accumulation (70, 71, 72, 73). Treatment of siCtrl RKO cells with LiCl induced GS3Kβ phosphorylation, activation of β-catenin, an increase in caspase-2 cleavage, and γH2AX accumulation, but not activation of p53 (Fig. 6, *E* and *F*). The level γH2AX was similar in the absence of caspase-2 and/or p53. In contrast, treatment with both lithium chloride and CPT resulted in stabilization of p53 and induction of caspase-2 cleavage. Similar to CPT-treated HCT116 cells, in the absence of both caspase-2 and p53, the level of γH2AX was significantly decreased compared to control-treated cells. While the difference we observed was not as pronounced as the HCT116 cell line, this is likely due to the presence of residual caspase-2 and p53 in the RKO cell line compared to the complete knockouts in the HCT116 cell line. This result suggests that inducing β-catenin activation is sufficient to convert RKO cells to be more similar to HCT116 cells with respect to the regulation of DNA repair by caspase-2 and p53.

Treatment of the HCT116 cells with LiCl also resulted in increased pGS3Kβ(S9). However, due to the constitutive activation of β-catenin, the levels of β-catenin remained relatively the same (Fig. 6, *G* and *H*). In contrast, the combination of LiCl and CPT induced γH2AX, that was reduced in the absence of both caspase-2 and p53 even further than what was seen with CPT alone. Unexpectedly, under these conditions, the total level of active β-catenin went down. This result indicates that LiCl likely further increases β-catenin to a threshold level that triggered a negative feedback loop. LiCl treatment did not fully recapitulate the reciprocal compensation of CPT-induced caspase-2 and p53 activation in RKO cells. Instead p53 activation was decreased in all cell types compared to those treated with CPT alone. This is most likely the result of the inhibition of GS3Kβ, which is known to positively regulate p53 independent of β-catenin (74, 75). However, while variable, across three experiments, caspase-2 cleavage was significantly increased in p53-deficient cells treated with LiCl plus CPT compared to CPT alone or LiCl alone. Together, these results strongly suggest that β-catenin activation facilitates the DNA repair that is regulated by caspase-2 and p53 and the reciprocal compensation between caspase-2 and p53.

### Loss of caspase-2 alters the transcriptional profile of p53 target genes

**Figure 7:**
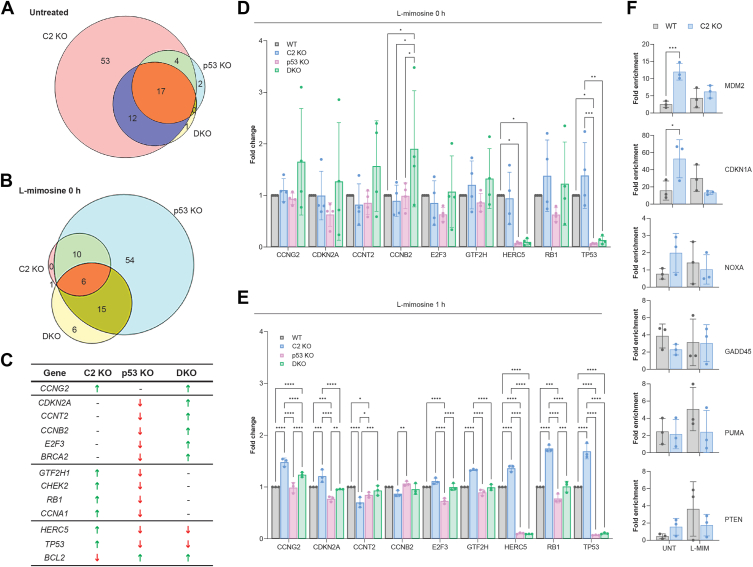
**Loss of caspase-2 alters the transcriptional profile of p53 target genes.***A–B,* HCT116*TP53*^+/+^/CRISPR scramble (WT), HCT116*TP53*^+/+^/CRISPR caspase-2 (C2 KO), HCT116*TP53*^−/−^/CRISPR scramble (p53 KO), and HCT116*TP53*^−/−^/CRISPR caspase-2 (DKO) cells were left untreated (*A*) or treated with L-mimosine (0.5 mM) for 22 h (0 h) (*B*). Gene expression of an array of 96 cell cycle genes was measured by real-time PCR. The number of genes with a >2× fold increase or >0.5-fold decrease for each genotype compared to WT are depicted in the Venn diagrams (Supplemental Data 1). *C,* differentially expressed genes shared between two or more of the knockout genotypes from the L-mimosine group are shown as static (−), upregulated (*green arrow*), or downregulated (*red arrow*). *D–E,* HCT116 WT, C2 KO, p53 KO, and DKO cells were treated with L-mimosine (0.5 mM) for 22 h (0 h) followed by removal of the treatment and incubation fresh media for 1 h. Gene expression of select genes from (*C*) was measured by real-time PCR at the 0 h (*D*) and 1 h (*E*) time points. ∗*p* < 0.05, ∗∗*p* < 0.01, ∗∗∗*p* < 0.001, ∗∗∗∗*p* < 0.0001 (Two-way ANOVA with Tukey’s multiple comparison test). *F,* HCT116 WT and C2 KO cells were treated as in (*A*) and enrichment of the selected genes at the p53 response elements was measured by ChIP qPCR. Results are the average of three independent experiments plus or minus standard deviation. ∗*p* < 0.05, ∗∗*p* < 0.01, ∗∗∗*p* < 0.001, ∗∗∗∗*p* < 0.0001 (Two-way ANOVA with Tukey’s multiple comparison test).

Given that the presence or absence of caspase-2 alters the ability of p53 to regulate the G1 checkpoint in HCT116 cells, we next set out to determine if any p53 targets are altered by caspase-2. Thus, we used real-time PCR to measure the expression of an array of 96 genes involved in the cell cycle. Following this, we separated the genes that had a greater than 2-fold increase in expression or greater than 0.5-fold decrease in expression compared to wildtype HCT116 cells and compared the genes across the genotypes. If the loss of caspase-2 were to have no effect on p53-dependent gene expression, we would expect that the p53 KO differentially regulated genes would completely overlap with the p53/C2 DKO differentially regulated genes but not with C2 KO cells. Somewhat unexpectedly, we did not observe this effect in untreated cells or in cells arrested in G1 with L-mimosine (Fig. 7, *A* and *B*). In untreated cycling cells, the largest transcriptional change was in the absence of caspase-2 compared to WT (86/96), with around a third of these (33/96) also being p53 dependent (Fig. 7*A*). After treatment with L-mimosine, the expression of the majority of the genes (85/96) was altered in the absence of p53 as expected, but only 21 of these genes were also differentially regulated with the additional absence of caspase-2 (Fig. 7*B*). This result suggests that the changed expression of 54 genes in the absence of p53 alone requires the presence of caspase-2. None of the genes were exclusively downregulated or upregulated in the arrested C2 KO cells, but there was an overlap of 16 genes with the p53 KO cells. These data indicate that caspase-2 contributes to p53-dependent gene expression.

We then focused on the G1 arrest-induced differentially regulated genes in common between the cell lines (Fig. 7*C*). *CCNG2* was the single gene that was upregulated in both C2 KO and p53/C2 DKO cells but not in p53 KO cells. Of the 15 genes shared between p53 KO p53/C2 DKO cells, five were decreased in the absence of p53 and increased in the absence of p53 and caspase-2, suggesting that loss of caspase-2 rescued the expression. Similarly, in genes that were shared between p53 KO and C2 KO cells, expression was decreased without p53 and increased without caspase-2 in 4 out of 10 genes. This is likely due to the increased expression of p53 upon G1 arrest. Finally, of the six genes that were differentially regulated in all three cell lines, the expression of half of these genes was altered in opposite directions in the presence and absence of p53.

From the list of differentially expressed genes, we chose nine to validate. We treated HCT116 cells with the G1 arrest inducer, L-mimosine, and measured gene expression by real-time PCR at the 0 h and 1 h time points following washout of the drug. At the 0 h time point, we observed a general upregulation of *CCNG2*, *CDKN2A*, *CCNT2*, and *CCNB2* in DKO compared to WT cells consistent with the results in Figure 7*C*, but the reduction in the absence of p53 alone was not as consistently evident (Fig. 7*D*). Of these, only *CCNB2* showed a significant upregulation in DKO cells compared to the other genotypes. *RB1* trended toward the pattern of increased expression in C2 KO and decreased expression in p53 KO. *HERC5* and *TP53* were both decreased in p53KO and DKO cells. *E2F3* and *GTF2H* did not match the pattern in Figure 7*C*. At the 1 h time point, the differences were more significant and matched the data in Figure 7*C* (Fig. 7*E*). *CCNG2* was upregulated in C2 KO and DKO cells but not in p53 KO cells. *CDKN2A* was increased without caspase-2, decreased without p53, and unchanged in the absence of both. *CCNT2* was decreased in C2 KO only. *E2F3* was deceased only in the absence of p53. *RB1* was increased without caspase-2, decreased without p53, and unchanged in the absence of both. *HERC5* and *TP53* were both increased in the absence of caspase-2 but decreased in the p53 KO and DKO cells indicating that p53 expression is transcriptionally repressed by caspase-2.

To determine how caspase-2 is influencing the p53 transcriptional profile, we measured p53 binding to response elements of common target genes in the presence and absence of caspase-2. p53 was enriched at the response elements for each gene in both untreated and L-mimosine-treated cells, and in most cases, the enrichment increased slightly with treatment (Fig. 7*F*). In untreated cells, binding of p53 to the response elements of *MDM2* and *CDKN1A* (the gene that encodes p21) was increased in the absence of caspase-2. The binding of p53 to the response elements of *NOXA* and *PTEN* also tended toward being increased in untreated C2 KO cells, but it was not significant. Loss of caspase-2 had no impact on the binding of p53 to the response elements of *PUMA* and *GADD45*. Interestingly, following treatment, loss of caspase-2 had minimal effects on the ability of p53 to bind to its response elements, and where there were differences, it trended toward reducing the binding efficiency (*CDKN1A*, *PUMA*). Overall, these data indicate that caspase-2 impairs binding of p53 to certain promoter regions in quiescent cells, likely explaining the altered transcriptional profile in the absence of p53.

## Discussion

Caspase-2 has been shown to function as a tumor suppressor in some cancer types and in a pro-oncogenic fashion in others (4, 5, 6, 7, 8, 9). However, what decides these differing functions is yet unclear. Here we showed that even in cell types with an intact p53 pathway that are derived from similar cancers, caspase-2 can have different functional outcomes, and the interplay between caspase-2 and p53 is also cell-type dependent. In particular, in the HCT116 colon cancer cell line, caspase-2 can compensate for the loss of p53, and p53 can compensate for the loss of caspase-2 in the regulation of cell division and HR. As a result, the absence of both proteins is particularly disadvantageous to the cell, resulting in impaired DNA repair and slower tumor growth.

**Figure 8:**
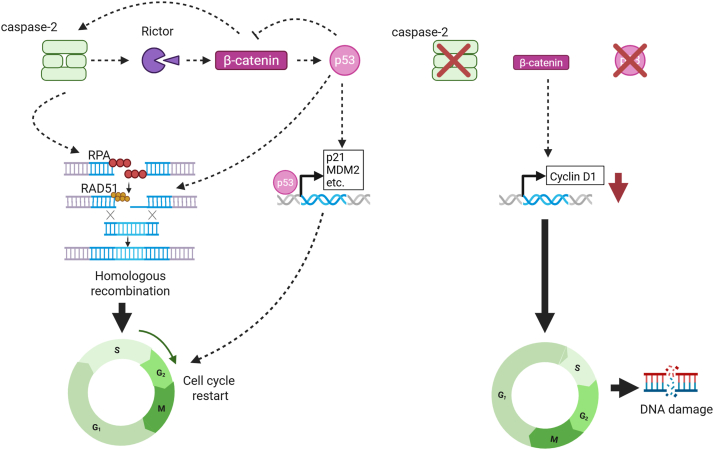
**Model of reciprocal compensation of caspase-2 and p53 activation.** See text for details.

In this study, we noted that high *CASP2* expression correlated with poorer outcomes in patients with colon cancer. This association partially stratified by *TP53* status with the worst outcomes in the group expressing high *CASP2* and mutant *TP53.* Consistent with these data, in chicken egg xenografts of the colon cancer cell line HCT116, tumor growth positively correlated with caspase-2 expression. Providing a possible explanation for these observations, we found that caspase-2 loss in HCT116 cells resulted in increased p53 activation and p53 loss increased caspase-2 activation. These data suggest that there are reciprocal compensatory pathways of these two proteins in this cell type. The removal of both proteins resulted in impaired proliferation, slower recovery from G1 arrest, reduced HR, and a disruption of the p53-dependent transcriptional program (Fig. 8). Surprisingly, *TP53* status alone was not sufficient to account for different responses of these cell lines as we did not see the same phenomenon in A375 melanoma and RKO colon cancer cells that are wildtype for *TP53.* Together, our data indicate a cell type-specific intricate relationship between caspase-2 and p53 acting as safeguards for the loss of each other in the regulation of the DDR.

The cell type-specific functions of caspase-2 have resulted in many conflicting results in the field, both concerning functions of caspase-2 in apoptosis and in cell cycle events (3). A recent report showed that, in hematopoietic cells with extra centrosomes, caspase-2 induces apoptosis primarily through BID cleavage, but if BID is absent, then caspase-2 will cleave MDM2, and apoptosis will proceed in a p53-dependent manner, whereas epithelial cells are more likely to undergo caspase-2- and p53-dependent cell cycle arrest under similar conditions unless BID is overexpressed (76). In many cell types, caspase-2 is antiproliferative and has a tumor suppressor function, while in neuroblastoma, caspase-2 loss protects from tumor progression, and in AML cells expressing mutated NPM1, caspase-2 is proproliferative (8, 11). We and others have previously reported that caspase-2 is important for cell cycle regulation in HeLa cells and in E1A/Ras-transformed MEF, cell types with defective p53 pathways (7, 10). However, the loss of p53 alone is not sufficient to explain these phenotypes as even in cells with wildtype p53 the functions of caspase-2 can be quite different. Instead, these differences might be determined by the involvement of other pathways.

These differences could be attributed to the pool of substrates available for caspase-2. While we failed to see cleavage of BID and cleavage of MDM2 alone could not explain the differences in p53 activation, we detected a caspase-2-dependent cleavage of the mTORC2 protein Rictor in the HCT116 cells. Rictor has been proposed as a caspase-2 substrate in neurons (50) and in NPM1c+ AML cells (11). Cleavage of Rictor removes the phosphorylation domain that prevents it from activating AKT and therefore potentially promotes the ability of Rictor to activate AKT (11, 77). Rictor is known to regulate gene expression and has been shown to bind to and inactivate wildtype p53 in hepatocellular carcinoma cells (78, 79). Thus, it is possible that the repressive effect of caspase-2 on p53 is due to Rictor activation induced by caspase-2-dependent cleavage. Supporting this idea, RKO colon cancer cells that did not show the reciprocal relationship between caspase-2 and p53 and showed minimal evidence of caspase-2-dependent Rictor cleavage. Thus, Rictor may be the key substrate that regulates these caspase-2 functions.

We have previously shown that loss of caspase-2 increases sensitivity to DNA damage in E1A/Ras-transformed MEF (10). In some of our experiments in HCT116 cells, we noted an increase in γH2AX in the absence of caspase-2. While we cannot rule out that the increase in p53 activation is a result of this increased DNA damage, this increase was not consistent across experiments, and it is equally possible that the increase of p53 activity increased γH2AX as the DNA damage response was initiated. Rather, our data suggest that the mechanism underlying whether reciprocal compensation between caspase-2 and p53 occurs is determined, at least in part, by the basal level of β-catenin activity. Unlike the RKO cells, HCT116 cells harbor a mutation in β-catenin resulting in high levels of activated WNT signaling (80). In NPM1c+ AML cells, loss of caspase-2 resulted in impairment of β-catenin activation that was associated an overall decrease in cell proliferation (11). Consistent with these observations, activation of β-catenin in CPT-treated RKO cells led to caspase-2- and p53-dependent γH2AX accumulation that was similar to that of CPT treatment in HCT116 cells. Therefore, the difference in baseline β-catenin activation in the two cell lines appears to underlie the DNA repair functions of caspase-2 and p53. Many types of colon cancer have both increased WNT signaling and p53 inactivation (81). Hyperactive β-catenin has been shown to lead to p53 accumulation (82), which, in turn, can downregulate β-catenin (83). In addition, β-catenin has been shown to promote caspase-2 expression in HCT116 cells. Therefore, the lower threshold in these cells for caspase-2 and p53 activation may have helped to uncover their compensatory roles. It is also possible that p53-mediated downregulation of β-catenin is partly responsible for reducing the activation of caspase-2 (Fig. 8). In addition, Rictor can regulate the WNT pathway through its activation of AKT and subsequent inhibition of GSK3β, which leads to derepression of β-catenin (70, 84). Therefore, caspase-2-mediated activation of Rictor may feed into the WNT pathway. While this would be expected to increase β-catenin and p53, it is possible that when β-catenin is already high, the negative feedback loop from p53 leads to a net repression of β-catenin and consequent reduction of p53. Indeed, when we treated HCT116 cells with a β-catenin activator plus CPT, the overall level of active β-catenin reduced. Our results suggest that the presence of both caspase-2 and p53 maintain β-catenin activity at a threshold level in cells where it is hyperactive. According to this model, in the absence of p53, increased caspase-2 activity promotes expression of β-catenin target genes like Cyclin D1 that can compensate for the loss of p53. In the absence of both p53 and caspase-2, Cyclin D1 expression is reduced, which would allow progression through the cell cycle checkpoints and a failure to repair DNA breaks. While our work here takes the first steps toward understanding the cross regulation of these three pathways, a number of knowledge gaps remain as to how caspase-2 regulates β-catenin activity and the specific role of Rictor. It will be important to further explore the role of WNT signaling following caspase-2 activation to fully understand the interconnected roles of WNT and p53 in colon cancer progression.

Although a number of studies have shown that caspase-2 has roles in regulating the cell cycle and proliferation, the exact stage of the cell cycle upon which caspase-2 has the most impact and its specific function during cell division has been unclear (85). We previously reported that caspase-2 has a potential role in regulating the intra-S phase checkpoint (10), while other papers have indicated a G2 checkpoint function (7, 86). In HCT116 cells, we see the largest effect of loss of both caspase-2 and p53 on recovery from G1 arrest, similar to *NPM1c*+ AML cells that undergo G1 arrest in the absence of caspase-2 (11). In this study, we used L-mimosine to induce arrest. Published work suggests that L-mimosine induces G1 arrest independent of p53 (87), but our data suggest that the arrest only appears p53-independent because caspase-2 is repressing p53. Following arrest by L-mimosine, p53 and caspase-2 both appear to be activated but keep each other in check with respect to G1 arrest ultimately allowing cells to bypass the G1 checkpoint. Our cell cycle data are corroborated by our observations that loss of caspase-2 enhances the ability of p53 to bind to the response elements of the genes encoding p21 and MDM2 but not p53 targets that are involved in cell death like PUMA and NOXA. MDM2 and p21 both play critical roles in the G1 checkpoint (38, 88), and our data suggest that caspase-2 prevents the binding of p53 to the promoter regions of these and possibly other genes altering the transcriptional profile of these cells. Thus, caspase-2 may have a role in normal cycling cells, preventing spurious activation of p53 during cell division.

Here, we report that both caspase-2 and p53 are required for intact HR in HCT116 cells. This is somewhat contrary to published work showing that p53 represses HR by transcriptionally regulating the expression of the RAD51 or by directly binding to RAD51, RAD54, and RPA in a transcriptional-independent manner (21, 22, 25). During HR, RPA recognizes and binds to single strand DNA that appears at resected ends of DSBs. RPA activates RAD51, which displaces the RPA from the DNA, searches for homology, and initiates the synaptic phase of HR. p53 has been shown to enhance RAD51 binding to DNA *in vitro* during the synaptic phase, and this interaction is thought to promote fidelity of DNA repair (89, 90). Indeed, p53 has been shown to promote HR in response to topoisomerase-I inhibition, which may explain our results (23, 24). PCNA is recruited during the postsynaptic phase to initiate extension of the nascent DNA strand (58). Our results show that both caspase-2 and p53 are required for γH2AX and RAD51 recruitment to DSBs and *PCNA* expression, but RPA recruitment was primarily impacted by the loss of caspase-2 alone. The presence of γH2AX on DSBs is required for RAD51 recruitment but is dispensable for RPA recruitment which may explain these results (91, 92). Together our results indicate that caspase-2 and p53 independently activate HR by promoting expression and recruitment of downstream proteins like RAD51, events that are suppressed in the absence of both proteins. The net outcome of this reciprocal inhibition results in cell cycle progression and DNA repair that is more permissive for tumor growth than without both proteins (Fig. 8). Together with our observations that caspase-2 impacts the transcriptional output of p53, our data suggest that by repressing p53 accumulation in response to DNA damage signals, caspase-2 impacts both transcriptional and nontranscriptional functions of p53.

Mutations in *TP53* are common, especially in human colorectal cancer (93). Because of this, therapies targeting the p53 pathway are not always successful. Most of the therapies targeting p53 have focused on activating wildtype *TP53* by disrupting its interaction with its negative regulator MDM2 (94). However, these compounds ultimately result in acquired resistance and can be toxic to normal tissue (95, 96, 97, 98, 99). In addition, these target therapies require wildtype *TP53*; therefore, in the case of tumors with mutated *TP53* like those found in colorectal cancer, the rescue of p53 would be a better approach. However, due to the plethora of *TP53* mutations, one single compound is not sufficient to overcome this problem (100). Because our results show that caspase-2 inhibition promotes p53 function, caspase-2 inhibition may be an effective therapy to enhance the survival of patients with colon cancer subtypes where p53 is not functional. In addition, it may be possible to stratify patients based on the level of caspase-2 expression as well as *TP53* status. Of course, this approach would be limited to cancers where caspase-2 promotes rather than inhibits tumor growth, underscoring the need to determine the mechanisms of caspase-2 on proliferation in multiple tumor models with wildtype and mutated p53.

In conclusion, while it might be counterintuitive that these two proteins with similar functions are inhibiting one another, the cross inhibition of caspase-2 and p53 may be a way for the cell to prevent uncontrolled activation of either protein acting as a safeguard against an uncontrolled DDR. While HR-mediated DNA repair has clear beneficial effects, too much HR can lead to an accumulation of intermediates and genomic instability. As a multifunctional protein, it is important to understand how exactly caspase-2 is regulated in the DDR in different cell types to fully appreciate its function and potential as a drug target.

## Experimental procedures

### Chemicals and antibodies

The following antibodies were purchased from Cell Signaling Technologies: anti-caspase-2 (#2224); anti-p53 (7F5), anti-p-p53 (Ser15); anti-p21 (12D1); anti-CDT1 (D10F11); anti-geminin (E5Q9S); anti-Rad51 (F1G6C); anti-Rictor (53A2, 2114); anti-BID (2002); anti-non-phospho (active) beta-catenin (Ser33/37/Thr41) (D13A1); anti-GSK-3 beta (27C10); anti-phospho-GSK-3 beta (Ser9) (5B3); and anti-Cyclin D1 (E3P5S). Anti-caspase-2 (Cln 11B4, MAB3507) and anti-γH2AX (05-636, JBW301) antibodies were purchased from Millipore Sigma; anti-MDM2 (IF2, MA1-113), anti-p53 (DO-7, MA5-12557), and anti-caspase-2 (23GB4375, MA5-53732) antibodies were purchased from Thermo Fisher; anti-phospo RPA32 (S33, A300-246A) antibody was purchased from Fortis Life Sciences. All cell culture media reagents were purchased from Thermo Fisher. Cdk1 inhibitor (#21-769-95MG) was purchased from Thermo Scientific. Aphidicolin (#BML-CC101-0001) was purchased from Enzo. ZM 447439 (#S1103) and iCRT14 (#S8704) were purchased from Selleckchem. Unless otherwise indicated, all other reagents were purchased from Millipore Sigma.

### Cell culture and cell lines

HCT116, RKO, and A375 cells were grown in Dulbecco’s modified essential medium containing FBS (10% (v/v)), L-glutamine (2 mM), and penicillin/streptomycin (50 I.U/50 μg/ml). To generate HCT116 and A375 cells expressing HR and NHEJ reporters, cells were transfected with pimEJ5GFP (10 μg, Addgene #44026) (52) linearized XhoI or pHPRT-DRGFP (10 μg Addgene #26476) (53) linearized with Sac1 and Kpn1 and selected with puromycin (0.5 μg/ml). A375 and RKO cells stably expressing shp53 were generated by lentivirus transduction with pLVTH-sip53 (a gift from Didier Trono Addgene plasmid #12239 (ref: https://pubmed.ncbi.nlm.nih.gov/12885912/). To generate lentivirus, HEK 293T cells were transiently transfected with pLVTH-sip53 along with the packaging vector, pSPAX-2, and the envelope vector pVSV-G using Lipofectamine 2000 transfection reagent (Thermo Fisher Scientific) according to manufacturer’s instructions. After 48 h, virus-containing supernatants were cleared by centrifugation and incubated with A375 cell lines. To generate retrovirus, Gryphon Ampho/Phoenix Ampho cells were transiently transfected with pMX-p53ERtam-IRES-GFP (47, 48) using the Lipofectamine 2000 transfection reagent. After 48 h, virus-containing supernatants were cleared by centrifugation and added along with 5 μg/ml polybrene to HCT116*TP53*^−/−^/CRISPR caspase-2 (DKO) cells. Single cell clones were isolated, and expression of p53 was confirmed by Western blot and fluorescence. HCT116*TP53*^+/+^ and HCT116*TP53*^−/−^ were obtained from B.Vogelstein (36). The *TP53* gene was amplified from genomic DNA isolated from HCT116*TP53*^+/+^/CRISPR scramble (WT), HCT116*TP53*^+/+^/CRISPR caspase-2 (C2 KO), and A375 cells with the following primers:

hTP53_FL_For: ATGGCGACTGTCCAGCTTTGTGC

hTP53_FL_Rev: ACGGTTGGTTCCTGAGTTATGAATGG and The gene was sequenced to confirm that the *TP53* sequence was wildtype in each cell line. A375 and RKO cells were purchased from ATCC. Cell line authenticity was validated using STR profiling, and cell lines were validated to be *mycoplasma* free prior to use in experiments.

### CRISPR/Cas9 gene editing

The *CASP2* gene was deleted from HCT116 *TP53*^+/+^ and HCT116*TP53*^−/−^ cells using an adaptation of the protocol in ref (101). The *CASP2* gene was targeted using two sgRNAs, which were designed using www.crisprscan.org. DNA templates for sgRNAs were made by PCR using a pX459 plasmid (10 ng/μl) containing the sgRNA scaffold sequence, Phusion High fidelity master mix (New England Biolabs #M0531S), and the following primers (10 μM):

ΔCASP2(76) sequence: ttaatacgactcactataGGCGTGGGCAGTCTCATCTTgttttagagctagaaatagc;

ΔCASP2(73) sequence: ttaatacgactcactataGGTGTGGAGGGCGCCATCTAgttttagagctagaaatagc;

scramble: ttaatacgactcactataGGCGCGATAGCGCGAATATATTgttttagagctagaaatagc universal primer: AGCACCGACTCGGTGCCACT.

The sgRNAs were synthesized by *in vitro* transcription using the HiScribe T7 high yield RNA synthesis kit (New England Biolabs #E2040S), and RNA was purified using the RNA purification kit (Zymo Research #R1017). 0). Purified sgRNA (0.5 μg) was incubated with Cas9 (1 μg, PNA Bio #CP02) for 10 min at room temperature. HCT116 cells were electroporated with the sgRNA/Cas9 complex using the Neon Transfection system (Thermo Fisher) at 1530 V, 20 ms, one pulse. Single-cell clones were isolated, and knockout was confirmed by Western blotting.

### Plasmids, siRNA, and transient transfection

pCBASceI (Addgene #26477) (102), pimEJ5GFP (Addgene #44026) (52), and pHPRT-DRGFP (Addgene #26476) (53) were purchased from Addgene. pcDNA3.1^(+)^ vector (#V79020) was purchased from Invitrogen, and caspase-2 full length was cloned into the pcDNA3.1^(+)^ vector. The pBiFC.VC155 and pBiFC.VN173 plasmids are available from Addgene (44). Control siRNAs were siCyclophilin B (ON-TARGETplus SMARTpool from Dharmacon). Caspase-2 siRNAs were ON-TARGETplus SMARTpool (Dharmacon Inc).

Cyclophilin B control pool target sequences:

ACAGCAAAUUCCAUCGUGU, GAAAGAGCAUCUACGGUGA, GAAAGGAUUUGGCUACAAA, GGAAAGACUGUUCCAAAAA.

Caspase-2 sequences: GCCUUGCACUCCUGAAUUU, UAGUCAAGGUGCUCAAUAA, CAUAGUGGGCCUUCAUUAA, UGAGAUAACUUCCUUCACA.

1 × 10^5^ cells were transfected with the appropriate plasmid or siRNA combinations using Lipofectamine 2000 transfection reagent (Thermo Fisher) according to manufacturer’s instructions. Cells were transfected with amounts of the relevant expression plasmids or siRNAs as described in the figure legends. Expression was allowed for 24 h, and media were exchanged for fresh media prior to treatment.

### Flow cytometry

All flow experiments were performed using an LSR Fortessa Flow Cytometer (BD). Data were analyzed using FlowJo Software (BD). For Annexin V binding, cells were treated as indicated in the figure legends. Cells were harvested by centrifugation at 300 × *g* for 5 min and washed with 1X PBS, and resuspended in 200 μl of Annexin V staining buffer (10 mM Hepes, 150 mM NaCl, 5 mM KCl, 1 mM MgCl2, and 1.8 mM CaCl2) containing 2 μl of Annexin V-APC (Thermo Fisher Scientific). After 15 min of incubation at room temperature, Annexin V-positive cells were quantified by flow cytometry. For cell cycle analysis, cells were treated as indicated in the figure legends and were stained using the BD Pharmingen FITC BrdU Flow Kit (BD Biosciences #559619). Following the treatment, cell medium was exchanged for medium with BrdU (10 μM). After 30 min, cells were harvested by centrifugation at 300 × *g* for 5 min and resuspended in 50 μl of BD cytoperm for 15 min in the dark. Cells were washed in BD perm/wash buffer with FBS and resuspended in 50 μl of BD Cytofix/Cytoperm buffer for 10 min covered on ice. Finally, the cells were resuspended in 50 μl of BD Cytoperm buffer for 5 min covered at room temperature. To expose the BrdU, cells were resuspended in 50 μl of DNase A (300 mg/ml) for 1 h in the dark. Cells were washed in BD perm/wash buffer and centrifuged at 300 x g and then incubated in a 1:50 dilution of anti-FITC-BrdU antibody for 20 min at room temperature. Cells were washed again and resuspended in 300 μl of (3% FBS in PBS) containing 10 μl 7-AAD/1 × 10^6^ cells. Cells were quantitated for BrdU and 7-AAD positivity by flow cytometry.

### Immunoblotting

The lysates were harvested in radioimmunoprecipitation assay buffer (150 mM NaCl, 50 mM Tris-HCl, pH 7–8, 0.1% SDS, 0.5% sodium deoxycholate, 1% IGEPAL) supplemented with plus complete protease inhibitors-EDTA (1 mini tablet/10 ml, Sigma 04693132001). Cleared protein lysates were resolved by SDS-PAGE on a 4 to 12% Bis-Tris gradient gel (Thermo Fisher NP0321BOX) and transferred into nitrocellulose membrane (Thermo Fisher # 88018). Membranes were incubated with the corresponding primary antibodies and peroxidase-coupled secondary antibodies (Genesee # 84-848, #84-854, or #20-307). Proteins were visualized with the AFP Mini Med 90 Xray Film Processor using West Dura and West Pico chemiluminescence substrate (Thermo Fisher Scientific # 34580 or # 34075). To quantitate protein expression in western blots, the intensity of each protein band and the background was measured with ImageJ (NIH). Proteins were normalized to the loading control loaded in each lane or, in the case of unphosphorylated proteins, to the total level of that protein.

### Viability assay

1.25 × 10^3^ HCT116 cells, RKO cells, or A375 cells transfected with the indicated siRNAs the prior day were plated per well in 96-well plates. Cell growth was measured daily for 4 days using the MTT cell proliferation kit (Millipore, 1146500700). The medium from each well was removed and replaced with fresh medium containing 10 μl of 12 mM MTT as per the manufacturer’s instruction. After 4 h of incubation, MTT medium was replaced by 100 μl of DMSO followed by orbital plate shaking. The plates were then incubated for an additional 15 min. The absorbance was read at 540 and 680 nm wavelengths.

### DNA repair assay

Cells stably expressing either pimEJ5GFP or pHPRT-DRGFP were transfected with pCBASceI in the presence or absence of siRNAs as indicated in the figure legends. After 3 days, the cells were collected in FACS buffer (1% BSA, 0.5 mM EDTA in PBS) and the percentage of cells, expressing mCherry and GFP were measured by flow cytometry.

### Real-time PCR

5 x 10^5^ HCT116*TP53*^+/+^/CRISPR scramble (WT), HCT116*TP53*^+/+^/CRISPR caspase-2 (C2 KO), HCT116*TP53*^−/−^/CRISPR scramble (p53 KO), and HCT116*TP53*^−/−^/CRISPR caspase-2 (DKO) were plated in 10-cm dishes and treated as indicated in the figure legends. RNA was extracted using the RNeasy kit (QIAGEN 74104). The RNA concentration was measured, and 1300 ng of RNA were used to make cDNA using the GoScript Reverse Transcriptase kit (Promega A2801). The cDNA was diluted 1:10, and expression was quantified using the TaqMan Fast Advanced Master Mix (Thermo Fisher #4444557) and the StepOne Real-Time PCR System (Thermo Fisher). The following Taqman probes (Thermo Fisher) were used: array of 96 cell cycle genes (TAQMAN ARRAY 96-WELL FAST PLATE 1X96, #4413255), RB1 (4453320 Hs01078066_m1), GTF2H1 (4448892 Hs00366525_g1), CCNA1 (4453320 Hs00171105_m1), CCNG2 (4453320 Hs00171119_m1), TP53 (4453320 Hs01034249_m1), HERC5 (4453320 Hs00180943_m1), CDKN2A (4453320 Hs00923894_m1), CCNB2 (4448892 Hs01084593_g1), CCNT2 (4448892 Hs00171034_m1), E2F3 (4453320 Hs00605457_m1), RAD51C (4331182 Hs04194939_s1), PCNA (4453320 Hs00427214_g1), and GAPDH (4453320 Hs02786624_g1).

### Chicken embryo CAM

Human colorectal cancer cells HCT116 were engrafted on the CAM for 7 days following previously established methods (103). Briefly, embryonic day 7 eggs were inoculated with 2.5 × 10^5^ cells in a 1:1 Matrigel and PBS (supplemented with calcium and magnesium) solution. Tumors were imaged, harvested, and weighed at day 7 postengraftment. Chick embryos were euthanized per AVMA guidelines. CAM tumors were fixed in 10% formalin and embedded in paraffin blocks for downstream immunohistochemical analysis. For lysates, frozen tumors were crushed using a mortar and pestle, and the tumors were resuspended in 250 μl of radioimmunoprecipitation assay buffer/10 mg of tumor followed by homogenization using a QIAshredder (QIAGEN # 79654).

### Histology

Sections were cut from each formalin-fixed paraffin-embedded block at 5 μm thickness and mounted on positively charged slides. One section was deparaffinized and stained with hematoxylin & eosin (Epredia) using standard histological methods. Ki-67 (Clone SP6) IHC was performed with heat-induced epitope retrieval and rabbit on rodent detection (Biocare). Pictures of the Ki-67 slides were taken using the Aperio CS2 slide scanner.

### Human data

Mutation and mRNA expression data from different types of cancer were downloaded from cBioPortal (https://www.cbioportal.org/) (33, 34, 35). The *CASP2* mRNA levels of patients with either *TP53* WT or *TP53* MT tumors were compared. The studies consisted of the following: skin cutaneous melanoma (TCGA, PanCancer Atlas); colorectal adenocarcinoma (TCGA, PanCancer Atlas); breast invasive carcinoma (TCGA, PanCancer Atlas); lung adenocarcinoma (TCGA, PanCancer Atlas); and ovarian serous cystadenocarcinoma (TCGA, PanCancer Atlas). Overall survival in months and survival status were downloaded from cBioPortal for the following studies: skin cutaneous melanoma (TCGA, PanCancer Atlas); colorectal adenocarcinoma (TCGA, PanCancer Atlas); breast invasive carcinoma (TCGA, PanCancer Atlas); lung adenocarcinoma (TCGA, PanCancer Atlas); and ovarian serous cystadenocarcinoma (TCGA, PanCancer Atlas). Survival of the patients was compared between patients with high *CASP2* mRNA levels (above the median) and low *CASP2* mRNA levels (below the median) and based on *TP53* status where indicated.

### ChIP qPCR

Isolation of the chromatin bound by p53 was done using the protocol in reference (104). Briefly, 5 × 10^6^ HCT116, HCT116 *CASP2*^*−/−*^, HCT116 *TP53*^*−/−*^, and HCT116 *CASP2*^*−/−*^*/TP53*^*−/−*^ cells were plated in 15-cm dishes and left untreated or treated with 0.5 mM L-mimosine for 22 h. The cells were fixed with 1% paraformaldehyde (Electron Microscopy Sciences, #15710) plus complete protease inhibitors-EDTA (1 mini tablet/10 ml, Sigma 04693132001) by rotating for 10 min. Glycine was added at a final concentration of 0.125 M, and the cells were rotated for 10 min. The cells were washed with cold PBS twice and resuspended in 600 μl of sonication buffer (50 mM Hepes pH 7.9, 140 mM NaCl, 1 mM EDTA, 1% Triton, 0.1% sodium deoxycholate, 1% SDS, and 1X complete protease inhibitors). The cells were sonicated using the Bioruptor Pico sonication device (Diagenode) in 100 μl aliquots doing 30 cycles at high frequency with 30 s OFF and 30 s ON. Chromatin fragmentation was verified by running a 1% agarose gel. The fragmentated chromatin was incubated with 1 μg/ml of the p53 antibody (DO-7, MA5-12557, Thermo Fisher) or 1 μg/ml of the mouse IgG isotype control antibody (02-6502, Thermo Fisher) rotating overnight at 4 °C. The chromatin was incubated with 30 μl of the Dynabeads Protein G beads (10-003-D, Fisher Scientific), previously washed three times with blocking buffer (0.5% BSA), for 4 h rotating at 4 °C. The supernatant was removed, and the beads were washed for 5 min rotating with the following buffers: low salt (0.1% SDS, 1% Triton, 2 mM EDTA, 20 mM Tris-HCl pH 8.1, and 150 mM NaCl), high salt (0.1% SDS, 1% Triton, 2 mM EDTA, 20 mM Tris-HCl pH 8.1, and 500 mM NaCl), LiCl (0.25 M LiCl, 1% sodium deoxycholate, 1 mM EDTA, 10 Mm Tris-HCl pH 8.1, 1% NP-40), and TE (10 mM Tris-HCl pH 8.1, 1 mM EDTA). The chromatin was eluted from the beads by adding 210 μl of elution buffer (50 mM Tris-HCl pH 8.1, 10 mM EDTA, 1% SDS, preheated at 65 °C) and incubating at 65 °C, vortexing the beads every 5 min. The supernatant was removed from the beads, and it was incubated in a 65 °C water bath for 16 h to reverse crosslinks. After 16 h, 210 μl of TE buffer, 200 mM NaCl, and 0.2 mg/ml Rnase A were added, and the chromatin was incubated at 37 °C for 2 h. Proteinase K was added to a final concentration of 0.2 mg/ml, and the chromatin was incubated at 37 °C for 2 h. The chromatin was purified using the QIAquick PCR Purification Kit for PCR Cleanup (28104, QIAGEN) and eluted in 50 μl of EB buffer. Binding of p53 to its response elements was done by qPCR. Primer used can be found in Table 1 (105, 106, 107).

**Table 1:** List of ChIP qPCR primers

Gene	Forward primer (5′ to 3′)	Reverse primer (5′ to 3′)
MDM2 (91)	GGGCAGGTTGACTCAGCTTTT	AGCTGGGAAAATGCATGGTTTA
CDKN1A (91)	AGCAGGCTGTGGCTCTGATT	CAAAATAGCCACCAGCCTCTTCT
NOXA (90)	GTCCAGCGTTTGCAGATG	AACGAGGTGGGAGGAGAA
PUMA (90)	GCGAGACTGTGGCCTTGTGT	CGTTCCAGGGTCCACAAAGT
GADD45A (90)	GGATCTGTGGTAGGTGAGGGTCAGG	GGAATTAGTCACGGGAGGCAGTGCAG
PTEN (92)	CAAAAGCCGCAGCAAGTG	GAGCGCAGAGTCCCCAAG

### Immunofluorescence and bimolecular fluorescence complementation

1 × 10^5^ HCT116*TP53*^+/+^/CRISPR scramble (WT), HCT116*TP53*^+/+^/CRISPR caspase-2 (C2 KO), HCT116*TP53*^−/−^/CRISPR scramble (p53 KO), and HCT116*TP53*^−/−^/CRISPR caspase-2 (DKO) cells were plated in 12-wells glass plates coated with 0.1 mg/ml poly-d-lysine (A3890401, Thermo Fisher). The cells were treated as stated in the figure legends and fixed with 4% paraformaldehyde (Electron Microscopy Sciences, #15710) for 10 min. The cells were washed three times with PBS containing calcium and magnesium and permeabilized with 0.15% (v/v) Triton for 10 min. The cells were blocked with 2% (w/v) BSA for 30 min and stained with anti-RAD51 (65653S Cell Signaling) and anti-RPA (A300-246A, Fortis Life Sciences) at a 1:100 dilution for 1 h. The cells were incubated with Goat anti-Rabbit IgG (H + L) Cross-Adsorbed Secondary Antibody, Alexa Fluor 555 (A-21428, Thermo Fisher) at a 1:250 dilution for 45 min and incubated with 400 nM SYTO 13 green (S7575, Thermo Fisher) in the dark for 1 h prior to imaging. For the BiFC assay, HCT116*TP53*^+/+^/CRISPR scramble (WT) and HCT116*TP53*^−/−^/CRISPR scramble (p53 KO) cells were plated in 12-wells plates at a density of 1 × 10^5^ cells per well. Caspase-2 activation was determined using the caspase-2 BiFC system previously described in (44). The cells were transfected with pBiFC.VC155 (10 ng), pBiFC.VN173 (10 ng), and DsRedmito (10 ng) as a transfection reporter. One day post-transfection, the cells were treated as stated in the figure. The following day, the percentage of cells that were DsRedmito^+^ and Venus^+^ was determined for at least 200 cells per treatment group. Cells were imaged using a spinning disk confocal microscope (Carl Zeiss MicroImaging), equipped with a CSU-X1A 5000 spinning disk unit (Yokogawa Electric Corporation), multilaser module with wavelengths of 458 nm, 488 nm, 514 nm, 561 nm, and 647 nm, and an Axio Observer Z1 motorized inverted microscope equipped with a precision motorized XY stage (Zeiss). Images were acquired with a Zeiss Plan-Neofluar 40 × 1.3 NA or 63 × 1.4 NA objective on an Orca R2 CCD camera using Zen 2.5 software (Zeiss).

### Statistical analysis

All statistical analysis were done using GraphPad Prism 10 for Windows (USA RRID: SCR_002798). Statistical tests are listed in the figure legends.

## Data availability

The survival and expression data are publicly available in cBioPortal (https://www.cbioportal.org/) (33, 34, 35). The studies used consisted of the following: Skin Cutaneous Melanoma (TCGA, PanCancer Atlas); colorectal adenocarcinoma (TCGA, PanCancer Atlas); Breast Invasive Carcinoma (TCGA, PanCancer Atlas); Lung Adenocarcinoma (TCGA, PanCancer Atlas); and Ovarian Serous Cystadenocarcinoma (TCGA, PanCancer Atlas).

## Supporting information

This article contains supporting information.

## Conflict of interest

The authors declare that they have no conflicts of interest with the contents of this article.
