# Supplementary material for: Reciprocal compensation of caspase-2 and p53 regulates the DNA damage response in HCT116 colon cancer cells
Source: J Biol Chem. 2026 May 27;302(7):113199. doi: 10.1016/j.jbc.2026.113199 (PMC13320034; doi:10.1016/j.jbc.2026.113199)
Supplement: Supporting Figures [file mmc2.docx]

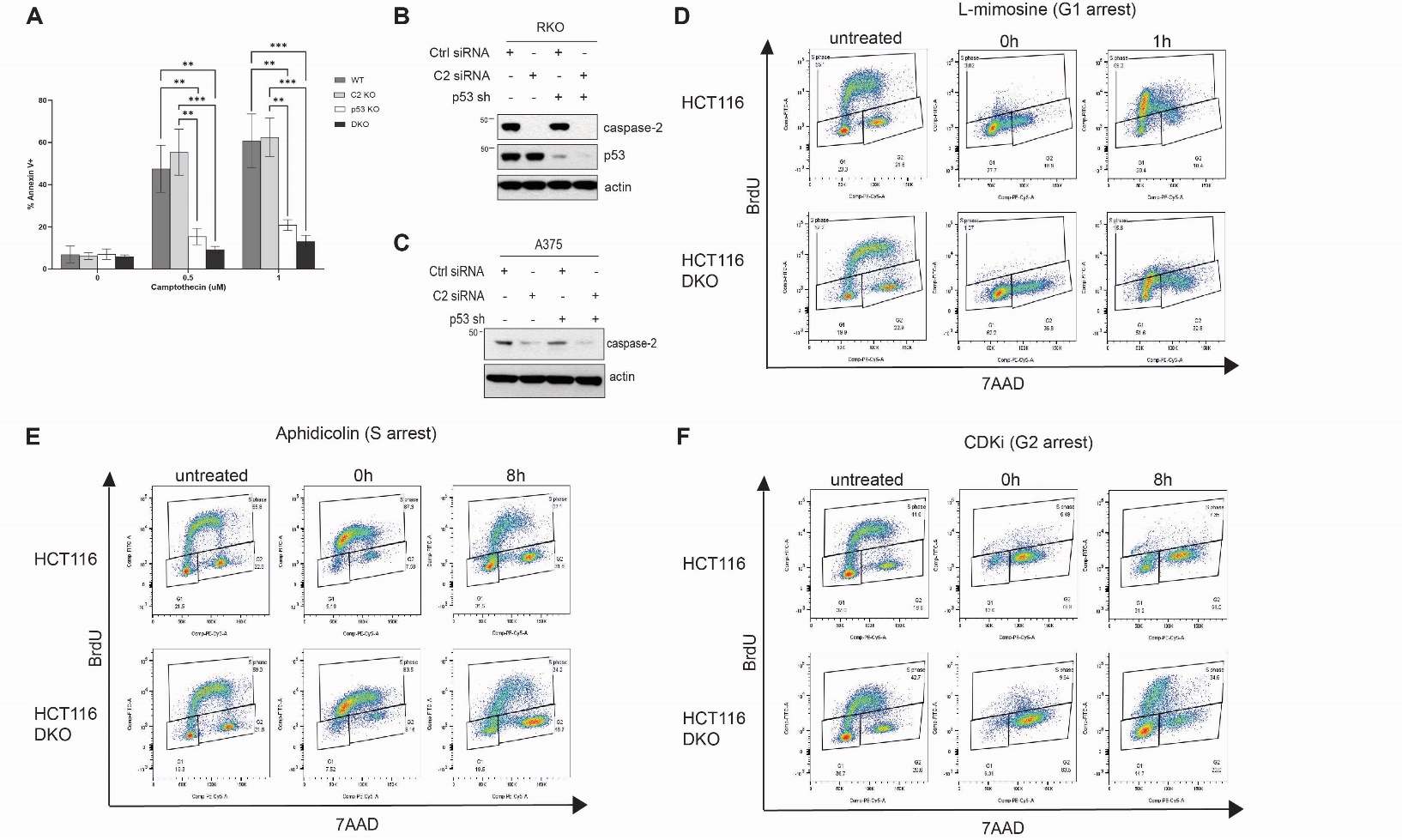


**Supplemental Figure S1. Validation of siRNA and representative flow plots** (matching Figure 3)**. A.** HCT116*TP53*^+/+^/parental (WT), HCT116*TP53*^+/+^/CRISPR caspase-2 (C2 KO), HCT116*TP53*^-/-^/parental (p53 KO), and HCT116*TP53*^-/-^/CRISPR caspase-2 (DKO) cells were left untreated or treated with the indicated amount of camptothecin for 16 h. Apoptosis was measured by flow cytometry for Annexin V binding. Results are the average of three independent experiments plus or minus standard deviation. *p < 0.05, ***p < 0.001 (One-way ANOVA with Dunnett’s multiple comparison test). **B-C.** RKO (B) and A375 (C) cells stably expressing an shRNA targeting p53 were transiently transfected with either siRNA against caspase-2 or a control siRNA (25 nM). Four days after the transfection the cell lysates were immunoblotted for the indicated proteins with actin as a loading control. Results are representative of three independent experiments. **D-F.** HCT116*TP53*^+/+^/CRISPR scramble (WT), and HCT116*TP53*^-/-^/CRISPR caspase-2 (DKO) cells were left untreated or treated with L-mimosine (0.5 mM) for 22 h (D), aphidicolin (1 µM) for 16 h (E), or double thymidine block followed by inhibition of CDK1 using Ro-3306 (CDK1i, 10 µM) for 12 h (F). The media was exchanged for fresh media (0h) and the cells were collected at the indicated time-points. The cells were pulsed for with BrdU (10 μM) 30 min prior to each time-point and then stained with anti-BrdU-FITC/7-AAD at the indicated time-points. Representative flow plots are shown of data quantitated in Figure 3D (L-mimosine), Figure 3E (aphidicolin), and Figure 3F (CDKi) respectively.


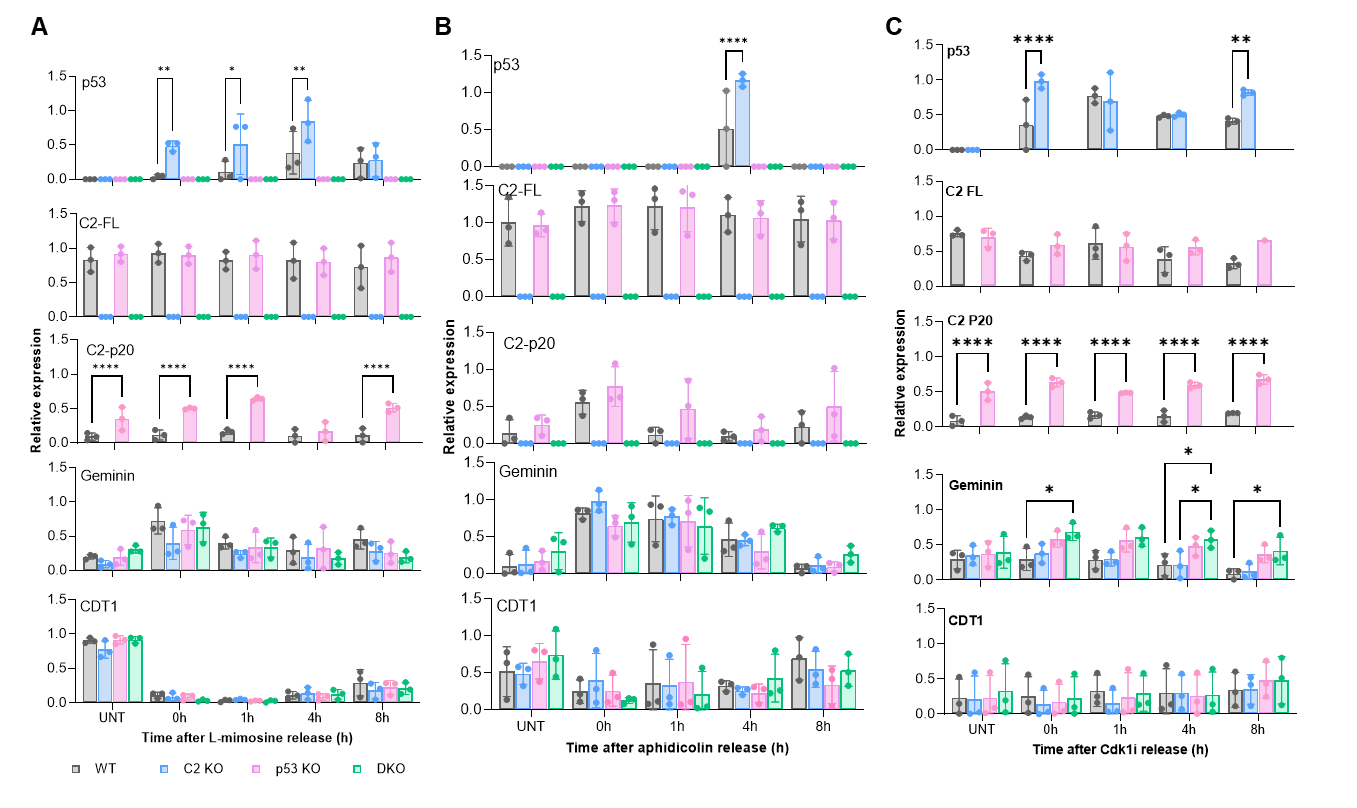


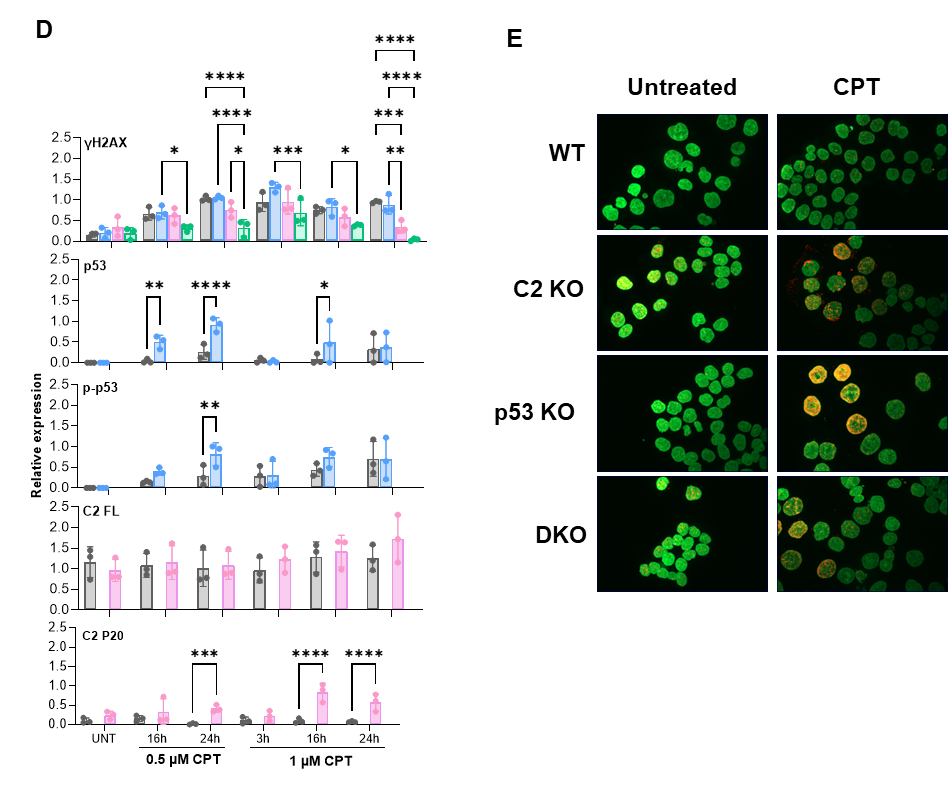


**Supplemental Figure S2: Quantification of western blots in Figure 4 A-E.**

**A-C.** HCT116*TP53*^+/+^/CRISPR scramble (WT), HCT116*TP53*^+/+^/CRISPR caspase-2 (C2 KO), HCT116*TP53*^-/-^/ CRISPR scramble (p53 KO), and HCT116*TP53*^-/-^/CRISPR caspase-2 (DKO) cells were left untreated or treated with L-mimosine (0.5 mM) for 22 h (A), aphidicolin (1 µM) for 16 h (B), double thymidine block followed by inhibition of CDK1 using Ro-3306 (CDK1i, 10 µM) for 12 h (C), or CPT for 3 h (D) followed by replacement of fresh media (0h). Cell lysates were collected at the indicated time-points after recovery and were immunoblotted for the indicated proteins with actin as a loading control. Band intensity of the proteins was quantified and normalized over the loading control actin. A-E represent quantitation of Figure 4A, 4B, 4C and 4E respectively. Results are the average of three independent experiments plus or minus standard deviation. *p < 0.05, **p < 0.01, ***p < 0.001, ****p < 0.0001 (Two-way ANOVA with Tukey’s multiple comparison test). (E) Representative γH2AX staining of cells treated as in (D).


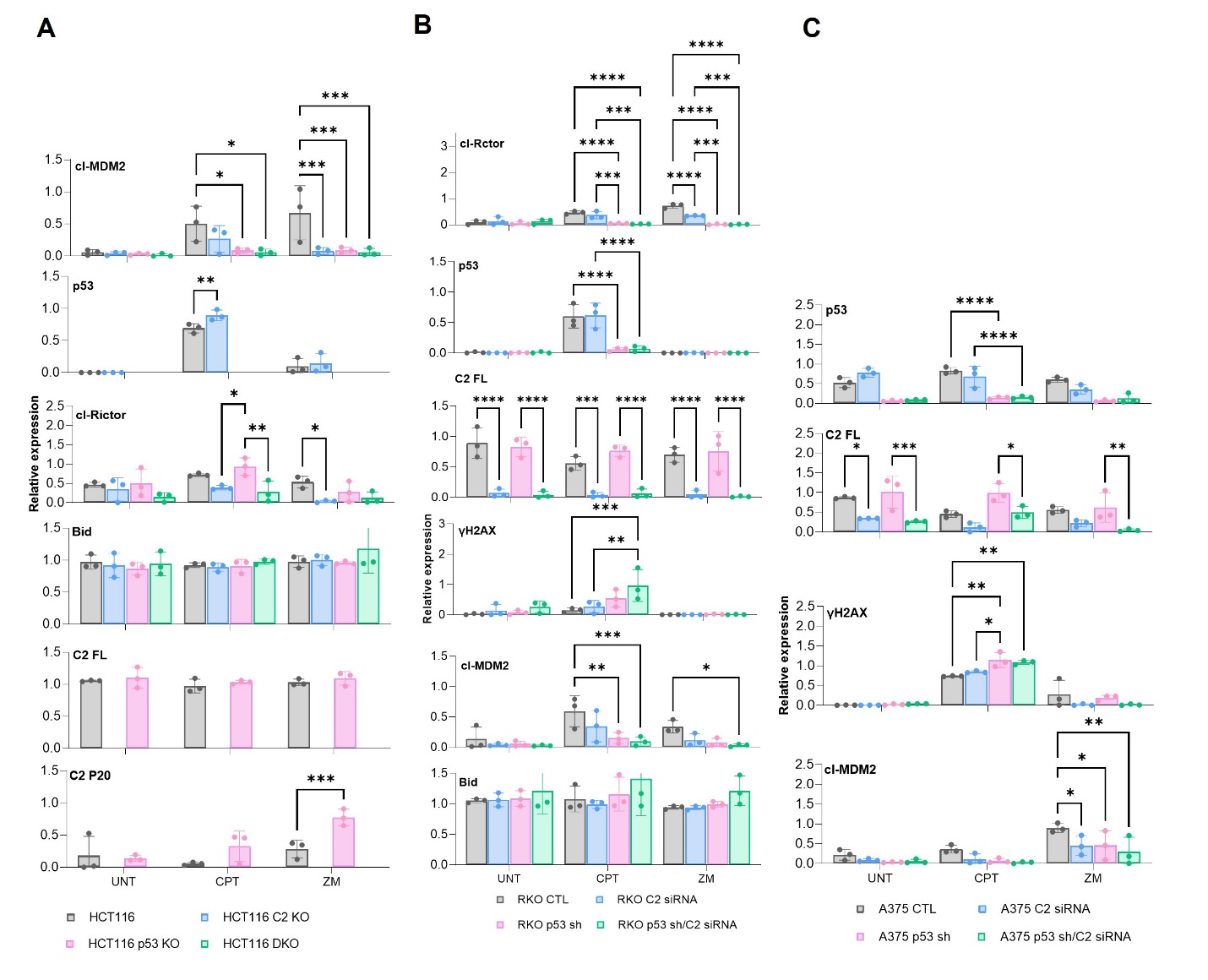


**Supplemental Figure S3. Quantification of western blots in Figure 4 H-J.**

**A.** HCT116*TP53*^+/+^/CRISPR scramble (WT), HCT116*TP53*^+/+^/CRISPR caspase-2 (C2 KO), HCT116*TP53*^-/-^/ CRISPR scramble (p53 KO), and HCT116*TP53*^-/-^/CRISPR caspase-2 (DKO) cells were left untreated or treated with CPT (0.5 µM) for 3 h followed by replacement with fresh media for 24 h, or with ZM447439 (ZM, 2 µM) for 16 h. Cell lysates were immunoblotted for the indicated proteins with actin as a loading control. **B-C.** RKO (B) or A375 (C) cells stably expressing an shRNA targeting p53 were transfected with either siRNA against caspase-2 or a control siRNA (25 nM). Two days after transfection, cells were treated as in (A) and cell lysates were immunoblotted for the indicated proteins with actin as a loading control. Band intensity of the proteins was quantified and normalized over the loading control actin. A-C represent quantitation of Figure 4H, 4I, and 4J respectively. Results are the average of three independent experiments plus or minus standard deviation. *p < 0.05, **p < 0.01, ***p < 0.001, ****p < 0.0001 (Two-way ANOVA with Tukey’s multiple comparison test).


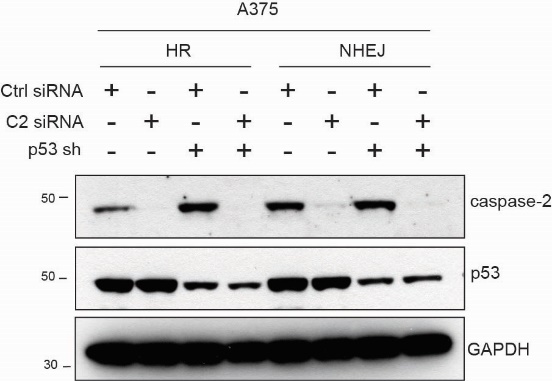


**Supplemental Figure S4. Validation of siRNA in A375 cells** (matching figure 5)**.** A375 cells stably expressing an shRNA targeting p53 and pDRGFP (HR) or pimEJ5GFP (NHEJ) were transiently transfected with I-SceI (100 ng), H2B-mCherry (150 ng) as a transfection reporter, and either siRNA against caspase-2 or a control siRNA (25 nM). Three days after the transfection the cell lysates were immunoblotted for the indicated proteins with actin as a loading control. Results are representative of three independent experiments.
